# Ropivacaine Versus Bupivacaine in Pediatric Tonsillectomy: A Systematic Review and Meta‐Analysis

**DOI:** 10.1002/oto2.70166

**Published:** 2025-09-19

**Authors:** Ebraheem Albazee, Khaled Alenezi, Abdulwahab Alkandari, Abdullah Al Sahli, Faisal Almulla, Khalaf A. Alnowaishiri, Athari Alwael

**Affiliations:** ^1^ Otorhinolaryngology‐Head and Neck Surgery Kuwait Institute for Medical Specializations (KIMS) Kuwait City Kuwait; ^2^ Department of Otolaryngology‐Head and Neck Surgery Al‐Jahra Hospital Al‐Jahra Kuwait; ^3^ Department of Surgery, Faculty of Medicine University of Jordan Amman Jordan; ^4^ Department of Internships Kuwait Institute for Medical Specializations (KIMS) Kuwait City Kuwait

**Keywords:** analgesia, bupivacaine, pain, ropivacaine, tonsillectomy

## Abstract

**Objective:**

To evaluate the analgesic efficacy and safety of local anesthetic infiltration in the tonsillar fossa using ropivacaine compared to bupivacaine in pediatric patients undergoing tonsillectomy.

**Data Sources:**

CENTRAL, PubMed, Web of Science, Scopus, and Google Scholar.

**Review Methods:**

Eligible randomized controlled trials (RCTs) were evaluated for risk of bias using Cochrane's Risk of Bias Tool (RoB‐2). The primary outcome was postoperative pain within the first 24 hours following tonsillectomy. Secondary outcomes included the time to first analgesic requirement and complication rates (ie, bleeding, airway obstruction, local anesthetic toxicity, and nausea). Data were synthesized using the standardized mean difference (SMD) for continuous outcomes and risk ratio (RR) for dichotomous outcomes, both reported with 95% confidence intervals (CI).

**Results:**

Seven RCTs with a total of 375 patients were analyzed. Regarding posttonsillectomy pain scores, there was no significant difference between ropivacaine and bupivacaine at 1 hour (SMD = −0.01, 95% confidence interval [CI] [−0.36, 0.34]), 2 hours (SMD = 0.03, 95% CI [−0.45, 0.51]), 4 hours (SMD = −0.17, 95% CI [−0.39, 0.06]), 6‐8 hours (SMD = 0.04, 95% CI [−0.38, 0.46]), and 12 hours (SMD = −0.23, 95% CI [−0.62, 0.15]). However, at 24 hours, ropivacaine demonstrated a superior effect compared to bupivacaine (SMD = −0.23, 95% CI [−0.43, −0.03]). There was no significant difference between ropivacaine and bupivacaine in terms of time to first analgesia and complication rates (*P* > .05).

**Conclusion:**

This meta‐analysis demonstrated that ropivacaine and bupivacaine offer comparable clinical analgesic efficacy and safety profiles in pediatric patients undergoing tonsillectomy.

Tonsillectomy is a common surgical procedure for conditions such as recurrent tonsillitis and obstructive sleep apnea in both pediatric and adult populations.^1^ The decision for surgery depends on symptom severity, recurrence, and the patient's overall health.^1^ While generally safe, tonsillectomy carries risks, including pain, bleeding, nausea, and dehydration, with postoperative pain being a major concern.^2^, ^3^ Despite various analgesic strategies—such as local anesthetics, opioids, NSAIDs, and acetaminophen—pain management remains suboptimal, highlighting the need for improved approaches.^2^

In pediatric patients, the immediate postoperative period—particularly the first 12 to 24 hours—is a critical window for effective pain management. This timeframe is especially important in the context of outpatient same‐day tonsillectomy, where early discharge is common and adequate analgesia is essential for minimizing complications.^4^, ^5^ Poorly controlled pain during this period can lead to reduced oral intake, dehydration, sleep disturbances, and unplanned hospital readmissions.^6^, ^7^ While opioids may offer effective pain relief, their adverse effects, including sedation and respiratory depression, often limit their use.^8^ This has led to increasing interest in safer, long‐acting alternatives such as local anesthetics to manage early postoperative pain and support recovery in children undergoing tonsillectomy.

Local anesthetics, including lidocaine, bupivacaine, and ropivacaine, are commonly used for peritonsillar infiltration to enhance postoperative analgesia.^9^ Bupivacaine, a long‐acting amide‐type anesthetic, provides analgesia for up to 8 hours but carries a risk of cardiotoxicity at elevated serum levels, including ventricular arrhythmias and myocardial depression.^10^, ^11^ Ropivacaine offers comparable or longer analgesia—up to 12 hours—and has a more favorable safety profile, particularly with reduced central nervous system and cardiac toxicity.^10^, ^12^

Previous meta‐analyses have confirmed the individual efficacy of both agents: Sun et al demonstrated the safety and effectiveness of bupivacaine compared to placebo,^13^ and Albazee et al reported similar findings for ropivacaine.^14^ However, while both agents are widely used, head‐to‐head randomized controlled trials (RCTs) directly comparing bupivacaine and ropivacaine have produced inconsistent results.^9^, ^15^, ^16^, ^17^, ^18^, ^19^, ^20^ Moreover, no meta‐analysis to date has systematically examined their comparative efficacy and safety.

Given their pharmacologic differences and potential clinical implications, especially regarding toxicity and duration of action in the critical first 24 hours after tonsillectomy, a direct comparison is warranted. This study aims to fill that gap by systematically evaluating the analgesic efficacy and safety of ropivacaine versus bupivacaine in pediatric patients undergoing tonsillectomy. By synthesizing the available evidence, we aim to inform clinical decision‐making and optimize pain management strategies in routine pediatric otolaryngologic practice.

## Methods and Materials

Prior to the review process, this study was registered on the International Prospective Register of Systematic Reviews (PROSPERO) under the CRD ID: [CRD420251032253]. The systematic review and meta‐analysis were conducted following the guidelines of the Preferred Reporting Items for Systematic Reviews and Meta‐Analyses (PRISMA)^21^ statement and the Cochrane Handbook for Systematic Reviews of Interventions.^22^ Ethical approval was not required for this type of research.

### Eligibility Criteria

The inclusion criteria for this study were established using the PICO framework. Participants included pediatric patients (<18 years old) undergoing tonsillectomy, with or without adenoidectomy, for recurrent tonsillitis and/or obstructive sleep apnea. The intervention involved ropivacaine infiltration at the tonsillar fossa, regardless of dose and regimen, while the comparison group consisted of those receiving bupivacaine infiltration at the tonsillar fossa, also irrespective of dose and regimen. The outcomes of interest encompassed both primary and secondary endpoints, specifically postoperative pain within 24 hours, time to first analgesia, and complication rates, including bleeding, airway obstruction, local anesthetic toxicity, and nausea. Only prospective RCTs were eligible for inclusion.

Studies were excluded if they involved procedures other than tonsillectomy, such as uvulopalatopharyngoplasty, or if they examined interventions or comparisons other than ropivacaine and bupivacaine, such as tramadol or lidocaine. Additionally, study designs other than RCTs, including case reports, observational studies, review articles, conference abstracts, and letters, were not considered for inclusion.

### Search Strategy and Databases

We conducted a systematic search across 4 major databases—PubMed, Scopus, Web of Science, and the Cochrane Central Register of Controlled Trials (CENTRAL)—from their inception, with the latest update in March 2025, to identify relevant studies. The search strategy utilized a combination of terms related to tonsillectomy and local anesthetics, including various synonyms and chemical names for ropivacaine and bupivacaine, ensuring comprehensive coverage of the literature. Detailed search strategies for each database are provided in Supplemental Table S1, available online. No filters based on the country or language of publication were specifically applied during the search process.

To maximize the completeness of our review and minimize the risk of overlooking pertinent studies, we manually screened the reference lists of all included articles and explored additional sources such as ClinicalTrials.gov, the World Health Organization (WHO) Clinical Trials Registry, and ResearchGate. Furthermore, we attempted to contact study authors to obtain missing data or clarify key information.

### Selection Process and Screening

Two independent reviewers conducted a dual‐round evaluation of each study to assess its relevance and eligibility. The screening process was carried out in 2 stages: first, the titles and abstracts of all retrieved references were reviewed separately to determine their relevance to this meta‐analysis; second, the full‐text articles of selected abstracts were assessed for eligibility. Any disagreements between reviewers were resolved through discussion and consensus. The same approach was applied during the risk of bias and statistical assessment to ensure the accuracy and reliability of the included studies.

### Risk of Bias and Publication Bias

We utilized the Cochrane Risk of Bias Tool 2 (RoB‐2) to evaluate potential biases in the included RCTs.^23^ This tool assesses bias across several domains, including the randomization process, deviations from intended interventions, missing outcome data, outcome measurement, and selection of reported results. Based on these criteria, studies were classified as having a low risk, high risk, or some concerns regarding bias.

In this study, we were unable to assess publication bias using Egger's test for funnel plot asymmetry, as Egger et al^24^ have indicated that such an assessment is unreliable when fewer than 10 studies are pooled in a meta‐analysis.

### Data Items and Review Outcomes

A pilot data extraction was performed after retrieving the full texts of relevant studies to develop a structured Excel extraction form. The form was divided into 3 sections: summary characteristics of the included trials, baseline characteristics of the participants, and outcome data. The summary characteristics section captured key study details, including the first author's name, year of publication, country, study design, sample size, intervention details, method of tonsillectomy, timing of administration, and follow‐up duration. The baseline characteristics section recorded information on the number of patients in each group, age, gender, route of administration, and surgeon details.

The clinical outcomes assessed in this study included both primary and secondary endpoints. The primary outcome focused on evaluating postoperative pain within the first 24 hours using validated pain assessment tools, such as the Visual Analogue Scale (VAS),^9^, ^15^ Wong‐Baker Faces Scale (WBFS),^20^ and Modified Children's Hospital of Eastern Ontario Pain Scale (mCHEOPS).^16^ All these tools follow the same evaluation direction, where lower scores indicate less pain, and higher scores reflect greater pain intensity. Postoperative pain was analyzed at specific time intervals within the 24‐hour period, specifically at 1, 2, 4, 6–8, 12, and 24 hours following tonsillectomy.

The secondary outcomes included the time to first analgesic administration in cases of severe pain, regardless of the analgesic type used (ie, acetaminophen, NSAIDs, or opioids). Additionally, we assessed the complication rates, including bleeding (%), airway obstruction (%), local anesthetic toxicity (%), and nausea (%).

### Meta‐Analysis

The analysis of dichotomous data was performed using the Mantel‐Haenszel method, with results expressed as risk ratios (RR) accompanied by 95% confidence intervals (CI). For continuous data, the Inverse‐Variance method was employed, and the findings were summarized as standardized mean differences (SMD) with 95% CI. The use of SMD is particularly advantageous when pooling continuous data from trials that employed diverse assessment tools, as recommended by Cochrane guidelines.^22^ Given the variability in assessment methods and pain management protocols across studies, heterogeneity was anticipated. A random‐effects model was applied in all analyses to account for potential variability between studies. Significant heterogeneity was identified when the Chi‐square test yielded a *P *< 0.1 and the *I* ^2^ statistic exceeded 50%.^22^ To evaluate the robustness of the results, sensitivity analyses were conducted by sequentially excluding one RCT at a time and recalculating the effect size based on the remaining studies. Statistical significance was defined as a *P* < .05. All statistical analyses were performed using STATA software (version 18 for Windows). In cases where data were presented solely in graphical form, Web Plot Digitizer version 4 (Free Software Foundation) was utilized to extract the relevant numerical data. Subgroup analyses were performed based on the use of adrenaline.

## Results

### Systematic Review Search and Eligibility Process

**Figure 1:**
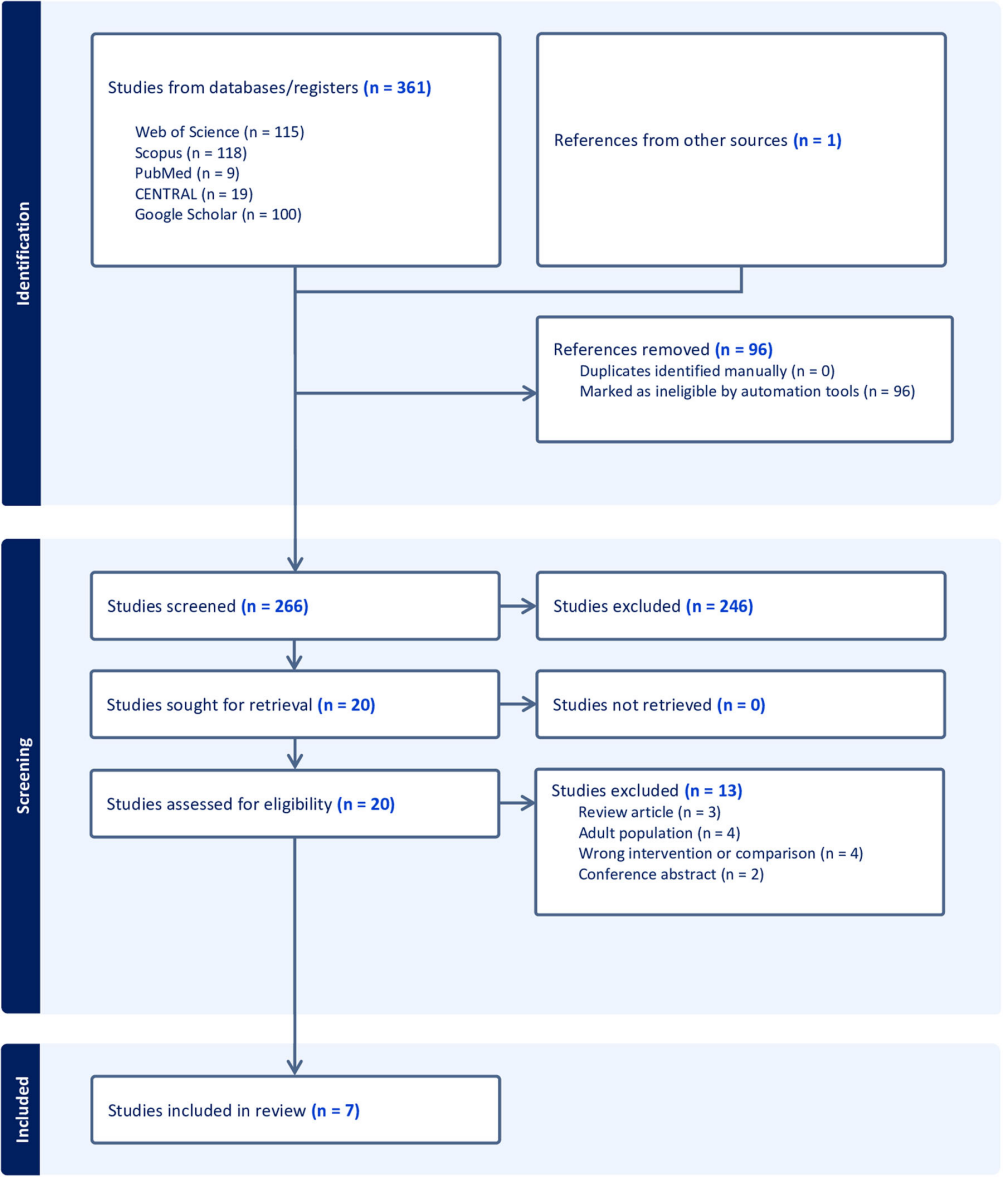
The preferred reporting items for systematic reviews and meta‐analyses (PRISMA) flow diagram illustrates the systematic study selection process.

The systematic search identified 361 studies from databases including Web of Science (n = 115), Scopus (n = 118), PubMed (n = 9), CENTRAL (n = 19), and Google Scholar (n = 100), with one additional reference from other sources. After removing 96 duplicates, 266 studies were screened. Of these, 20 were retrieved and assessed for eligibility, with 13 excluded due to being review articles, focusing on adult populations, having incorrect interventions, or being conference abstracts, as illustrated in Supplemental Table S2, available online. Ultimately, 7 RCTs^9^, ^15^, ^16^, ^17^, ^18^, ^19^, ^20^ met our PICO criteria, as depicted in Figure 1.

### Summary and Baseline Characteristics of the Included Trials

The included studies comprised a total sample size of 375 participants across 7 RCTs conducted in Turkey and India.^9^, ^15^, ^16^, ^17^, ^18^, ^19^, ^20^ The total number of males and females was 202 and 173, respectively. Among the participants, 188 received ropivacaine and 187 received bupivacaine. The timing of administration was consistently before tonsillectomy in all trials. The follow‐up periods varied, with most studies reporting outcomes at 24 hours, while others extended to 72 hours or 7 days. The studies compared different concentrations of ropivacaine and bupivacaine, with conventional tonsillectomy methods being the most common surgical approach. None of the included RCTs were self‐controlled or used a within‐subject design. Two RCTs used adrenaline as an adjuvant to both ropivacaine and bupivacaine,^15^, ^19^ one RCT used adrenaline only with the bupivacaine group,^9^ and 4 RCTs did not use adrenaline at all.^16^, ^17^, ^18^, ^20^ The summary and baseline characteristics of the included RCTs and participants are detailed in Tables 1 and 2. Different pain assessment tools and postoperative pain control protocols were used among the included RCTs, as shown in Supplemental Table S3, available online.

**Table 1:** Summary of the Details of the Included Trials

					Study arms					
Study ID	Country	Study design	Duration	Sample size, n	Intervention	Control	Method of tonsillectomy	Self‐control	Timing	Follow‐up
Akoglu 2006	Turkey	RCT	January 2005‐September 2005	31	0.2% ropivacaine	0.25% bupivacaine	Conventional	No	Before tonsillectomy	24 hours
Gudi 2014	India	RCT	Not reported	108	0.5% ropivacaine	0.5% bupivacaine	Not reported	No	Before tonsillectomy	24 hours
Kumar 2021	India	RCT	August 2019‐January 2020	40	0.2% ropivacaine	0.25% bupivacaine	Conventional	No	Before tonsillectomy	24 hours
Mehta 2019	India	RCT	Not reported	60	0.375% ropivacaine	0.25% bupivacaine	Conventional	No	Before tonsillectomy	72 hours
Özkıriş 2012	Turkey	RCT	March 2009‐April 2012	60	0.5% ropivacaine	0.25% bupivacaine	Conventional	No	Before tonsillectomy	7 days
Rao 2022	India	RCT	January 2021‐August 2022	36	0.2% ropivacaine	0.25% bupivacaine	Not reported	No	Before tonsillectomy	24 hours
Unal 2007	Turkey	RCT	Not reported	40	0.2% ropivacaine	0.25% bupivacaine	Electrocautery	No	Before tonsillectomy	24 hours

Abbreviation: RCT, randomized controlled trial.

**Table 2:** Baseline Summary of the Included Patients and Trials

Study ID	Group	Dose	Route	Sample size, n	Age (years) mean ± SD	Sex, n [male/female]	Adenoidectomy (with/without)	Surgeon
Akoglu 2006	Ropivacaine	0.2% (3‐5 ml)	Infiltration	15	6.1 ± 1.0	[10/5]	Without	ENT residents who had a similar level of experience
	Bupivacaine	0.25% (3‐5 ml)	Infiltration	16	6 ± 2.5	[10/6]		
Gudi 2014	Ropivacaine	0.5% (2 mg/kg with adrenaline 1:500,000)	Infiltration	54	11.59	[30/24]	With	Not reported
	Bupivacaine	0.5% (2 mg/kg with adrenaline 1:500,000)	Infiltration	54	11.76	[31/23]		
Kumar 2021	Ropivacaine	0.2% (3 ml per tonsil)	Infiltration	20	6‐12 (range)	[10/10]	With	ENT residents who had a similar level of experience
	Bupivacaine	0.25% (3 ml per tonsil)	Infiltration	20	6‐12 (range)	[5/10]		
Mehta 2019	Ropivacaine	0.375% (4 ml per tonsil)	Infiltration	30	7.28	[20/10]	Without	The same ENT surgeon
	Bupivacaine	0.25% (4 ml per tonsil)	Infiltration	30	7.75	[20/10]		
Özkıriş 2012	Ropivacaine	0.5% (2.5 ml solution injected to the tonsils)	Infiltration	31	7.75 ± 3.95	[16/15]	With	The same ENT surgeon
	Bupivacaine	0.25% with 1:200,000 epinephrine	Infiltration	29	8.15 ± 4.20	[16/13]		
Rao 2022	Ropivacaine	0.2% (3 ml)	Infiltration	18	Not available	[8/10]	With	Not reported
	Bupivacaine	0.25% (3 ml)	Infiltration	18	Not available	[8/10]		
Unal 2007	Ropivacaine	0.2% (3‐5 ml) with 1:200,000 epinephrin	Infiltration	20	7.2 ± 2.3	[14/6]	Without	The same ENT surgeon
	Bupivacaine	0.25% (3‐5 ml) with 1:200,000 epinephrin	Infiltration	20	7.5 ± 3.1	[18/2]		

Abbreviation: ENT, ear, nose, and throat.

### Risk of Bias Summary

**Figure 2:**
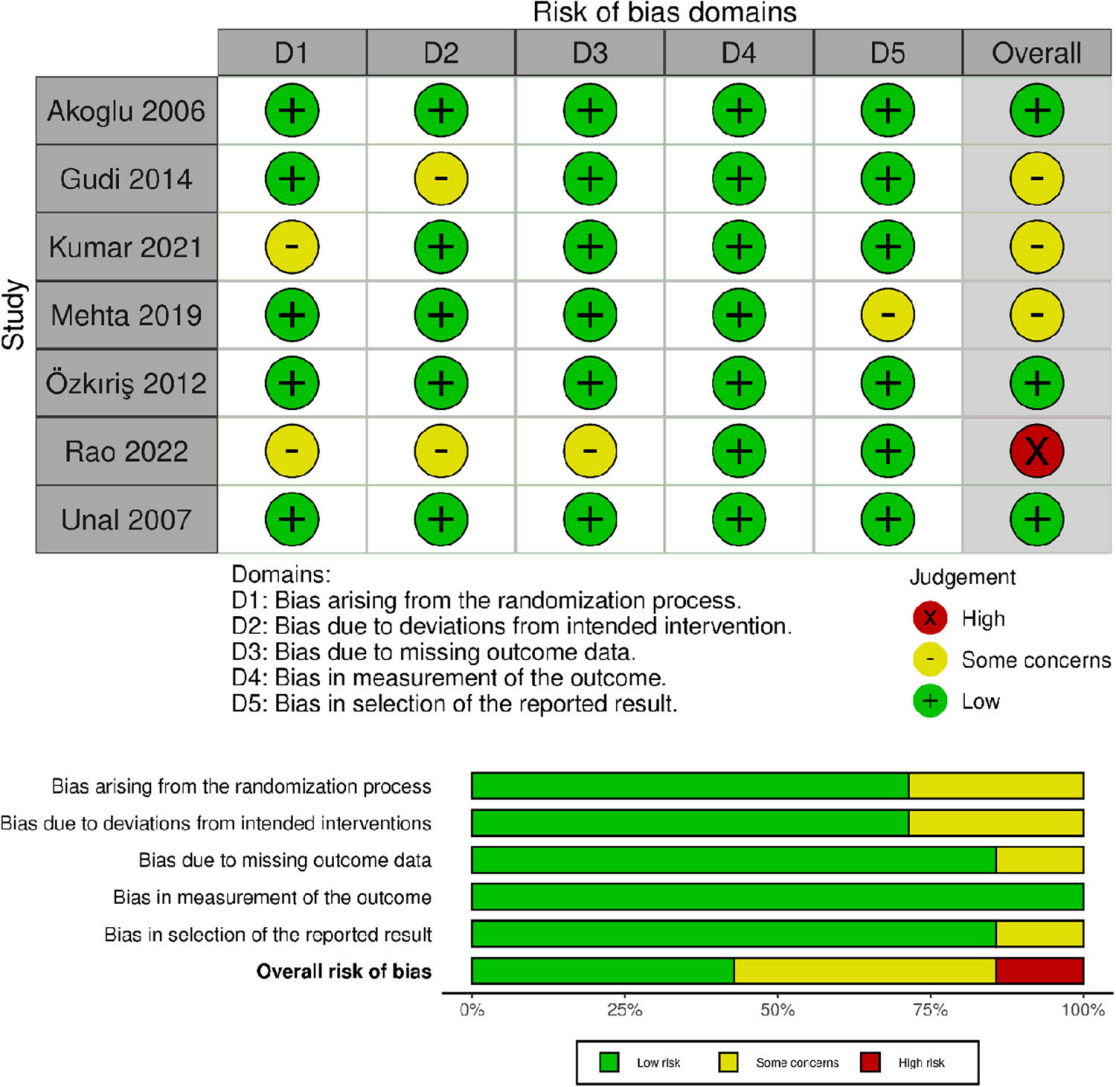
Risk of bias graph and summary of the included randomized controlled trials.

In summary, our assessment of the included studies revealed that 3 RCTs^9^, ^16^, ^19^ were deemed to have a “low” risk of bias, while 3 RCTs^15^, ^17^, ^20^ “some concerns” regarding bias. Additionally, 1 RCT^18^ was identified as having a “high” risk of bias, Figure 2. The study conducted by Gudi^15^ raised some concerns in the domain related to deviations from the intended intervention, as there was insufficient information to confirm if the surgeon and patients were blinded to the intervention or control. The study by Kumar^17^ raised concerns in the randomization process due to unclear methods of randomization and allocation concealment. Similarly, Mehta^20^ raised some concerns in the selection of reported results, potentially because it did not provide any data regarding complication rates as mentioned in the abstract. Lastly, the study by Rao^18^ identified as having a high risk of bias, primarily due to concerns and lack of information in the randomization process, deviations from intended interventions, and missing outcome data.

### Analgesic Efficacy

#### Postoperative Pain [1 hour]

**Figure 3:**
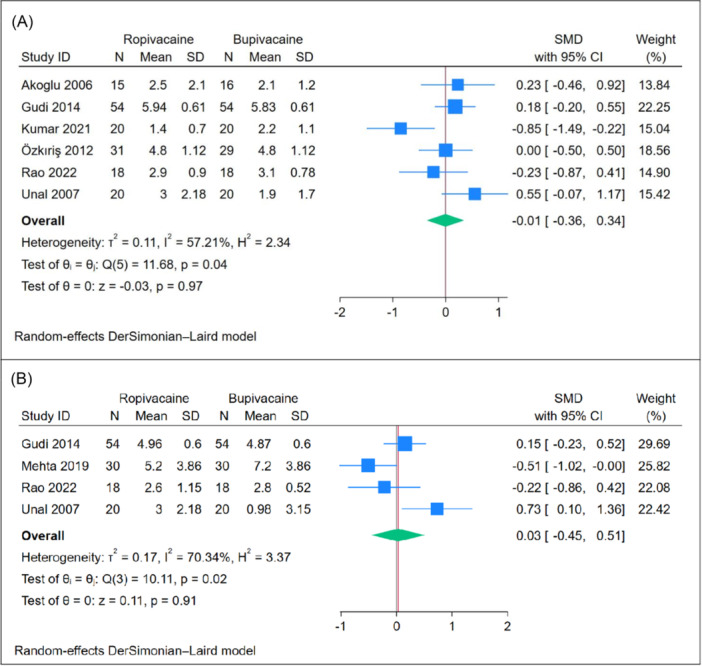
Meta‐analysis of mean postoperative pain scores: (A) at 1 hour and (B) at 2 hours. CI, confidence interval; SMD, standardized mean difference.

The mean pain score at 1 hour post‐tonsillectomy showed no significant difference between the ropivacaine and bupivacaine groups (n = 6 RCTs, SMD = −0.01, 95% CI [−0.36, 0.34], *P* = .97), as depicted in Figure 3A. The pooled analysis exhibited moderate heterogeneity (*P* = .04, *I* ^2^ = 57.21%). Leave‐one‐out sensitivity analysis demonstrated consistent results, with no significant difference between the two groups in all scenarios, as shown in Supplemental Figure S1A, available online. Subgroup analysis based on the use of adrenaline revealed no significant differences between the subgroups (*P* = .15), as illustrated in Supplemental Figure S1B, available online.

#### Postoperative Pain [2 hours]

The mean pain score at 2 hours posttonsillectomy showed no significant difference between the ropivacaine and bupivacaine groups (n = 4 RCTs, SMD = 0.03, 95% CI [−0.45, 0.51], *P* = .91), as depicted in Figure 3B. The pooled analysis exhibited moderate heterogeneity (*P* = .02, *I* ^2^ = 70.34%). Leave‐one‐out sensitivity analysis demonstrated consistent results, with no significant difference between the 2 groups in all scenarios, as shown in Supplemental Figure S2A, available online. Subgroup analysis based on the use of adrenaline revealed no significant difference between ropivacaine and bupivacaine when adrenaline was used (*P* = .18). However, ropivacaine demonstrated a superior effect compared to bupivacaine in studies where adrenaline was not used (*P* = .03), as illustrated in Figure 2B.

#### Postoperative Pain [4 hours]

**Figure 4:**
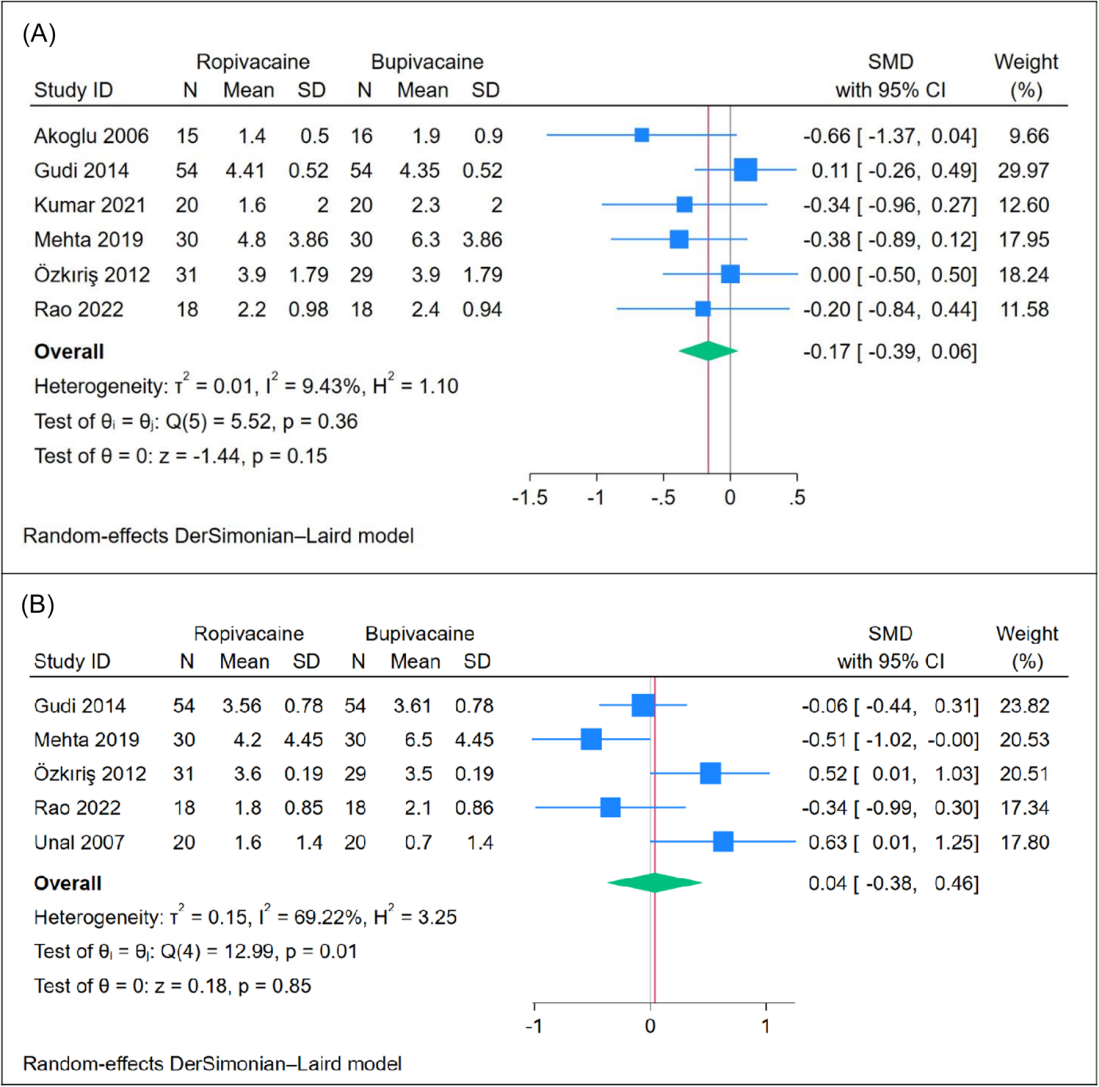
Meta‐analysis of mean postoperative pain scores: (A) at 4 hours and (B) at 6 to 8 hours. CI, confidence interval; SMD, standardized mean difference.

The mean pain score at 4 hours posttonsillectomy showed no significant difference between the ropivacaine and bupivacaine groups (n = 6 RCTs, SMD = −0.17, 95% CI [−0.39, 0.06], *P* = .15), as depicted in Figure 4A. The pooled analysis exhibited homogeneity (*P* = .36, *I* ^2^ = 9.43%). Leave‐one‐out sensitivity analysis demonstrated consistent results, with no significant difference between the two groups in most scenarios. However, when the study by Gudi^15^ was excluded, ropivacaine showed a superior effect compared to bupivacaine (*P* = .03), as shown in Supplemental Figure S3A, available online. Subgroup analysis based on the use of adrenaline revealed no significant difference between ropivacaine and bupivacaine when adrenaline was used (*P* = .63). However, ropivacaine demonstrated a superior effect compared to bupivacaine in studies where adrenaline was not used (*P* = .01), as illustrated in Figure 3B.

#### Postoperative Pain [6‐8 hours]

The mean pain score at 6 to 8 hours posttonsillectomy showed no significant difference between the ropivacaine and bupivacaine groups (n = 4 RCTs, SMD = 0.04, 95% CI [−0.38, 0.46], *P* = .85), as depicted in Figure 4B. The pooled analysis exhibited moderate heterogeneity (*P* = .01, *I* ^2^ = 69.22%). Leave‐one‐out sensitivity analysis demonstrated consistent results, with no significant difference between the two groups in all scenarios, as shown in Supplemental Figure S4A, available online. Subgroup analysis based on the use of adrenaline revealed no significant difference between ropivacaine and bupivacaine when adrenaline was used (*P* = .18). However, ropivacaine demonstrated a superior effect compared to bupivacaine in studies where adrenaline was not used (*P* = .03), as illustrated in Figure 4B.

#### Postoperative Pain [12 hour]

**Figure 5:**
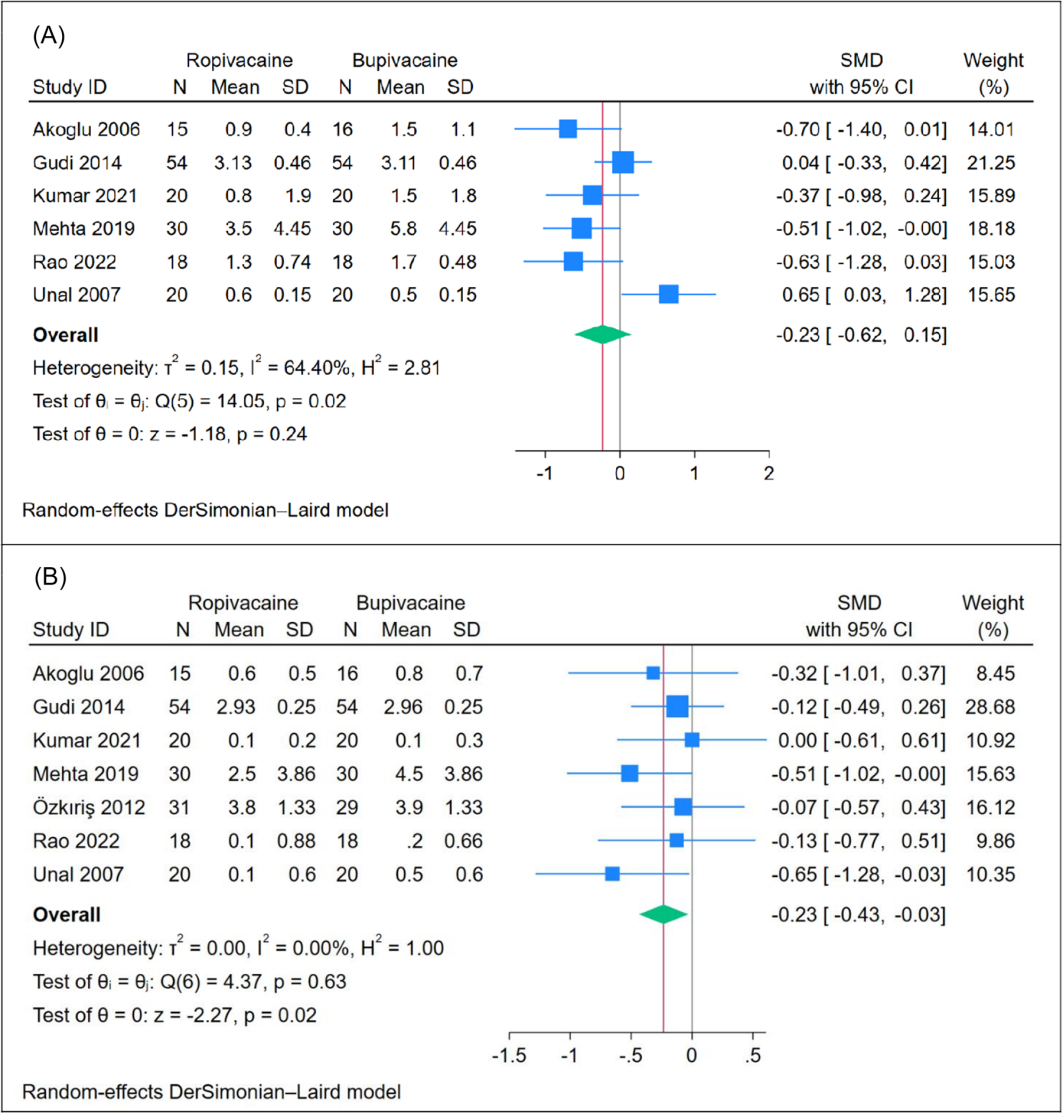
Meta‐analysis of mean postoperative pain scores: (A) at 12 hours and (B) at 24 hours. CI, confidence interval; SMD, standardized mean difference.

The mean pain score at 12 hours posttonsillectomy showed no significant difference between the ropivacaine and bupivacaine groups (n = 6 RCTs, SMD = −0.23, 95% CI [−0.62, 0.15], *P* = .24), as depicted in Figure 5A. The pooled analysis exhibited moderate heterogeneity (*P* = .02, *I* ^2^ = 64.40%). Leave‐one‐out sensitivity analysis demonstrated consistent results, with no significant difference between the two groups in most scenarios. However, when the study by Unal 2007^19^ was excluded, ropivacaine showed a superior effect compared to bupivacaine (*P* = .02), as shown in Supplemental Figure S5A, available online. Subgroup analysis based on the use of adrenaline revealed no significant difference between ropivacaine and bupivacaine when adrenaline was used (*P* = .33). However, ropivacaine demonstrated a superior effect compared to bupivacaine in studies where adrenaline was not used (*P* < .001), as illustrated in Figure 5B.

#### Postoperative Pain [24 hours]

The mean pain score at 24 hours posttonsillectomy indicated that ropivacaine demonstrated a superior effect compared to bupivacaine (n = 7 RCTs, SMD = −0.23, 95% CI [−0.43, −0.03], *P* = .02), as depicted in Figure 5B. The pooled analysis exhibited homogeneity (*P* = .63, *I* ^2^ = 0%). Leave‐one‐out sensitivity analysis showed that the significant difference between the two groups was lost when excluding the studies by Mehta^20^ and Unal,^19^ as shown in Supplemental Figure S6A, available online. Subgroup analysis based on the use of adrenaline revealed no significant differences between the subgroups (*P* = .83), as illustrated in Supplemental Figure S6B, available online.

### Time to First Analgesia

**Figure 6:**
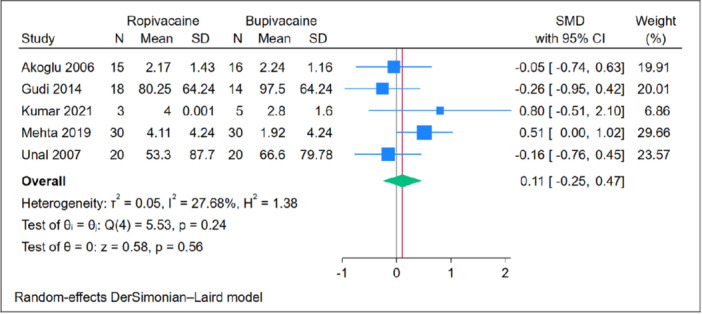
Meta‐analysis of mean score of time to 1st analgesia. CI, confidence interval; SMD, standardized mean difference.

The time to first analgesia showed no significant difference between the ropivacaine and bupivacaine groups (n = 5 RCTs, SMD = −0.11, 95% CI [−0.25, 0.47], *P* = .56), as depicted in Figure 6. The pooled analysis exhibited homogeneity (*P* = .24, *I* ^2^ = 27.68%). Leave‐one‐out sensitivity analysis demonstrated consistent results, with no significant difference between the two groups in all scenarios, as shown in Supplemental Figure S7A, available online. Subgroup analysis based on the use of adrenaline also revealed no significant differences between the subgroups (*P* = .08), as illustrated in Supplemental Figure S7B, available online.

### Safety Profile

Regarding the complication rate, there was no significant difference between ropivacaine and bupivacaine in terms of the rate of airway obstruction (n = 3 RCTs, RR = 1, 95% CI [0.11, 9.35], *P* = 1), hemorrhage (n = 3 RCTs, RR = 1, 95% CI [0.11, 9.35], *P* = 1), local anesthetic toxicity (n = 4 RCTs, RR = 1, 95% CI [0.41, 6.94], *P* = 1), and nausea (n = 2 RCTs, RR = 1.03, 95% CI [0.50, 2.12], *P* = .94), as depicted in Supplemental Figure S8, available online. The pooled analysis exhibited homogeneity (*P* = 1, *I* ^2^ = 0%).

## Discussion

In this systematic review and meta‐analysis, 7 RCTs comprising a total of 375 pediatric patients undergoing tonsillectomy were analyzed, with 188 receiving ropivacaine and 187 receiving bupivacaine. The methodological quality of the included studies varied: 3 RCTs were rated as low risk of bias, 3 had some concerns, and one was deemed to have a high risk of bias. Pain scores following tonsillectomy were generally comparable between the ropivacaine and bupivacaine groups across all assessed time points, except at 24 hours postoperatively, where ropivacaine was associated with a statistically significant reduction in pain. The time to first analgesic requirement was similar between groups. Both agents exhibited comparable safety profiles, with no significant differences in reported complications. Notably, in studies where adrenaline was used as an adjuvant, ropivacaine demonstrated a slight statistically significant advantage over bupivacaine at select time points (2‐12 hours).

Moreover, the included trials employed various pain assessment tools and postoperative analgesic protocols, which may explain the observed heterogeneity in some outcome measures. We used the SMD as the effect measure to address these differences and ensure that factors other than the local anesthetic agent (ropivacaine vs bupivacaine) did not confound the results. This approach, recommended by the Cochrane Collaboration when outcomes are measured using different scales, enabled us to standardize pain scores across studies and isolate the specific effect of the anesthetic agent used in each trial.^22^ Also, this method contributed to ensuring a robust and methodologically sound analysis.

Furthermore, the meta‐analysis revealed a considerable degree of heterogeneity. Several factors may account for this observation. The most prominent are differences in surgical technique, anesthetic dosing, and variations in perioperative analgesic protocols. Additional contributing factors include variability in patient age, surgical indication, and the experience level of the operating surgeon (consultant vs trainee). Methodological differences across studies and the use of diverse pain assessment tools may also explain the observed heterogeneity. These inconsistencies warrant cautious interpretation of the findings and underscore the need for more standardized study designs in future research.

Postoperative pain management remains a critical component of care in pediatric patients undergoing tonsillectomy.^2^ Adequate control of pain not only facilitates recovery but also reduces healthcare resource utilization and improves the overall patient experience.^2^ The American Academy of Otolaryngology–Head and Neck Surgery (AAO‐HNS)^25^ currently recommends against the routine use of local anesthesia in this context, citing insufficient evidence to support its efficacy in reducing postoperative pain.^25^, ^26^ This guidance is largely based on an earlier Cochrane review that assessed the use of local anesthetics but lacked a comprehensive quantitative synthesis.^26^ Since then, a growing body of high‐quality evidence—including several RCTs and meta‐analyses—has emerged, demonstrating both the safety and analgesic effectiveness of local anesthetic agents in pediatric tonsillectomy.^9^, ^13^, ^14^, ^16^, ^19^, ^27^, ^28^, ^29^, ^30^ Given these developments, it is increasingly important for future updates to clinical practice guidelines to re‐evaluate the current recommendation in light of the more robust and up‐to‐date evidence base.

The most commonly used local anesthetic in tonsillectomy has traditionally been 0.25% bupivacaine combined with 1:200,000 epinephrine, typically administered as a 3 to 5 ml injection into the peritonsillar tissue either before or after tonsil removal.^31^ However, alternative agents have also been explored, including 0.5% lidocaine with 1:100,000 epinephrine and ropivacaine.^9^ Compared to bupivacaine, ropivacaine is 2 to 3 times less lipid‐soluble and is characterized by a smaller volume of distribution, faster systemic clearance, and a shorter elimination half‐life.^32^ Notably, low concentrations of ropivacaine may produce clinically relevant vasoconstriction, which does not appear to be significantly enhanced by adding epinephrine.^32^, ^33^ Additionally, ropivacaine has been associated with a lower risk of cardiotoxicity compared to bupivacaine.^32^, ^33^ In the present meta‐analysis, ropivacaine demonstrated analgesic effects comparable to bupivacaine at all postoperative time points, except at 24 hours, where it showed a statistically significant advantage. However, the effect size (SMD = –0.23) was small and did not reach the minimal clinically important difference (MCID), generally considered greater than 1‐point, indicating that the difference was not clinically meaningful.^34^ Similarly, the time to the first analgesic requirement was comparable between the two groups. In line with these findings, Xu et al^35^ conducted a systematic review and meta‐analysis of 5 RCTs comparing intraperitoneal infiltration of ropivacaine and bupivacaine in laparoscopic cholecystectomy and reported comparable analgesic efficacy between the two agents. Our results are consistent with those of Xu et al,^35^ further supporting the conclusion that ropivacaine and bupivacaine provide similar postoperative pain control. While ropivacaine demonstrated a slight statistical advantage at the 24‐hour time point, this benefit did not translate into a clinically meaningful difference. Therefore, both agents appear to be equally effective options for postoperative pain management in pediatric tonsillectomy.

Interestingly, subgroup analysis revealed that ropivacaine demonstrated a slight advantage—less than 1 point on the pain scale—over bupivacaine at certain postoperative time points (2‐12 hours) when adrenaline was not used. In this context, Ozkiris et al^9^ conducted an RCT comparing ropivacaine without adrenaline to bupivacaine with adrenaline and found no statistically significant difference in mean postoperative pain scores between the 2 groups. Similarly, Gudi et al^15^ performed an RCT comparing ropivacaine with adrenaline to bupivacaine with adrenaline, concluding that both agents were equally effective in managing posttonsillectomy pain. More recently, Rao et al^18^ conducted an RCT comparing ropivacaine without adrenaline to bupivacaine without adrenaline. Their findings indicated that while both drugs were effective in reducing postoperative pain, ropivacaine provided superior analgesia compared to bupivacaine. All in all, these findings suggest that ropivacaine may offer a modest analgesic benefit over bupivacaine, particularly in the absence of adrenaline. Nonetheless, this potential advantage should be interpreted with caution, as the observed differences appear to be of limited clinical significance.

Bupivacaine, though widely used as a long‐acting local anesthetic, has been associated with cardiotoxic and neurotoxic effects, primarily due to its dextro enantiomer—raising safety concerns.^10^ Ropivacaine was developed as a safer alternative, offering similar analgesic duration with lower lipid solubility and reduced affinity for cardiac sodium channels, resulting in a more favorable toxicity profile.^32^, ^33^ Clinical evidence supports the safety of both agents when used for local infiltration in pediatric tonsillectomy, showing no increased risk of local anesthetic‐related toxicity compared to placebo.^13^, ^14^ In this meta‐analysis, ropivacaine and bupivacaine demonstrated comparable safety profiles across all assessed complications, including airway obstruction, hemorrhage, toxicity, and nausea, with no significant differences and complete homogeneity (*I* ^2^ = 0%). These findings further support the favorable and comparable safety profile of both agents in the pediatric tonsillectomy setting.

Although ropivacaine and bupivacaine demonstrated comparable safety and analgesic efficacy, ropivacaine is significantly more expensive.^36^ At The Mount Sinai Hospital in New York City, a 20‐ml vial of 0.2% ropivacaine costs $4.29, whereas a 30‐ml vial of 0.25% bupivacaine costs $1—making ropivacaine approximately ten times more costly per milligram.^36^ Given the absence of a clear clinical advantage, this substantial cost difference may limit the routine use of ropivacaine, especially in resource‐constrained settings.

The findings of this meta‐analysis hold practical relevance for otolaryngologists managing pediatric patients undergoing tonsillectomy. Although both ropivacaine and bupivacaine demonstrated favorable safety profiles and comparable analgesic efficacy, their benefits were primarily confined to the immediate postoperative period (ie, within the first 24 hours). Nevertheless, this timeframe is clinically important, as patients often experience the most discomfort during this phase and require effective pain control. Adequate management during this critical window is essential not only to improve patient comfort but also to reduce the risk of complications such as dehydration, poor oral intake, and unplanned hospital readmissions. Furthermore, effective early analgesia plays a key role in promoting recovery and facilitating a timely return to normal daily activities, including eating, sleeping, and communication—factors that are particularly important in the pediatric population.

This study has several notable strengths. It represents the first comprehensive systematic review and meta‐analysis comparing the analgesic efficacy and safety of ropivacaine versus bupivacaine in pediatric tonsillectomy. Only RCTs were included, and the methodology adhered to PRISMA and Cochrane guidelines. Additional strengths include the assessment of multiple outcomes and the use of robust statistical methods, such as subgroup and sensitivity analyses.

However, several limitations should be acknowledged. Most included studies had small sample sizes, and some outcomes exhibited high heterogeneity, likely due to differences in patient characteristics, surgical techniques, drug dosages, and pain assessment protocols. The generalizability of the findings may be limited, as all included studies were conducted in Turkey and India—regions that may differ from other countries in terms of clinical practice patterns, healthcare infrastructure, and access to specific anesthetic agents. These regional factors could influence outcomes and should be considered when applying the results to broader populations. Moreover, 3 studies were rated as having “some concerns” and one as “high” risk of bias. Finally, due to the small number of studies per outcome (<10), publication bias could not be reliably assessed, and funnel plot interpretations should be made with caution.

To address the limitations of this meta‐analysis, future research should prioritize large‐scale, multicenter RCTs with standardized surgical, anesthetic, and analgesic protocols to reduce heterogeneity and enhance generalizability. Ideally, such trials should focus on homogenous patient populations—such as children undergoing routine intracapsular tonsillectomy for obstructive sleep apnea or recurrent tonsillitis—and control for key variables including patient age, indication for surgery, surgical technique, and perioperative management. Future studies should also investigate the optimal dose, volume, and timing of ropivacaine and bupivacaine administration, including their use with or without vasoconstrictors (eg, epinephrine). Additionally, trials should incorporate clinically meaningful outcomes that were underreported in existing studies, such as postoperative narcotic requirements, hospital readmission rates, and time to return to normal activity, to better capture real‐world recovery. Investigations into the additive effects of combining these anesthetics with agents such as dexamethasone or ketamine may further clarify strategies to prolong analgesia and reduce postoperative inflammation. Finally, cost‐effectiveness analyses across diverse healthcare systems are essential to inform clinical practice and optimize resource allocation.

## Conclusion

This systematic review and meta‐analysis demonstrated that ropivacaine and bupivacaine offer comparable clinical analgesic efficacy and safety profiles in pediatric patients undergoing tonsillectomy. Further high‐quality, large‐scale RCTs are warranted to validate these findings and to explore the potential utility of ropivacaine and bupivacaine in combination with adjunctive agents.

## Author Contributions

**Ebraheem Albazee**, contributed to study conception, study design, data collection, data analysis, write up of original draft of manuscript, and review of manuscript for editorial and intellectual contents; **Khaled Alenezi**, contributed to study conception, study design, literature review, data collection, write up of original draft of manuscript, and review of manuscript for editorial and intellectual contents; **Abdulwahab Alkandari**, contributed to study conception, study design, literature review, data collection, write up of original draft of manuscript, and review of manuscript for editorial and intellectual contents; **Abdullah Al Sahli**, contributed to study conception, study design, literature review, data collection, write up of original draft of manuscript, and review of manuscript for editorial and intellectual contents; **Faisal Almulla**, contributed to study conception, study design, literature review, data collection, write up of original draft of manuscript, and review of manuscript for editorial and intellectual contents; **Khalaf A. Alnowaishiri**, contributed to study conception, study design, literature review, data collection, write up of original draft of manuscript, and review of manuscript for editorial and intellectual contents; **Athari Alwael**, contributed to study conception, study design, write up of original draft of manuscript, supervision and review of manuscript for editorial and intellectual contents; All authors read and approved the final draft of manuscript.

## Disclosures

### Competing interests

None.

### Funding source

None.

## Supporting information


**Figure S1. (A)** Leave‐one‐out sensitivity analysis; **(B)** Subgroup analysis based on the use of adrenaline for the mean postoperative pain score at 1 hour.
**Figure S2. (A)** Leave‐one‐out sensitivity analysis; **(B)** Subgroup analysis based on the use of adrenaline for the mean postoperative pain score at 2 hours.
**Figure S3. (A)** Leave‐one‐out sensitivity analysis; **(B)** Subgroup analysis based on the use of adrenaline the mean postoperative pain score at 4 hours.
**Figure S4. (A)** Leave‐one‐out sensitivity analysis; **(B)** Subgroup analysis based on the use of adrenaline for the mean postoperative pain score at 6‐8 hours.
**Figure S5. (A)** Leave‐one‐out sensitivity analysis; **(B)** Subgroup analysis based on the use of adrenaline for the mean postoperative pain score at 12 hours.
**Figure S6. (A)** Leave‐one‐out sensitivity analysis; **(B)** Subgroup analysis based on the use of adrenaline for the mean postoperative pain score at 24 hours.
**Figure S7. (A)** Leave‐one‐out sensitivity analysis; **(B)** Subgroup analysis based on the use of adrenaline for the mean score of time to 1^st^ analgesia.
**Figure S8.** Meta‐analysis of the rate of postoperative complications.
**Table S1.** Detailed search strategy for each database.
**Table S2.** List of excluded studies during the full‐text screening step.
**Table S3.** Detailed information regarding the assessment tool and pain control protocol for each trial.

## Data Availability

All data are available within the manuscript and can be obtained from the corresponding author upon a reasonable request.
