# Supplementary material for: Ropivacaine Versus Bupivacaine in Pediatric Tonsillectomy: A Systematic Review and Meta‐Analysis
Source: OTO Open. 2025 Sep 19;9(3):e70166. doi: 10.1002/oto2.70166 (PMC12447348; doi:10.1002/oto2.70166)
Supplement: Supplementary file 1 — Figure S1. (A) Leave‐one‐out sensitivity analysis; (B) Subgroup analysis based on the use of adrenaline for the mean postoperative pain score at 1 hour. Figure S2. (A) Leave‐one‐out sensitivity analysis; (B) Subgroup analysis based on the use of adrenaline for the mean postoperative pain score at 2 hours. Figure S3. (A) Leave‐one‐out sensitivity analysis; (B) Subgroup analysis based on the use of adrenaline the mean postoperative pain score at 4 hours. Figure S4. (A) Leave‐one‐out sensitivity analysis; (B) Subgroup analysis based on the use of adrenaline for the mean postoperative pain score at 6‐8 hours. Figure S5. (A) Leave‐one‐out sensitivity analysis; (B) Subgroup analysis based on the use of adrenaline for the mean postoperative pain score at 12 hours. Figure S6. (A) Leave‐one‐out sensitivity analysis; (B) Subgroup analysis based on the use of adrenaline for the mean postoperative pain score at 24 hours. Figure S7. (A) Leave‐one‐out sensitivity analysis; (B) Subgroup analysis based on the use of adrenaline for the mean score of time to 1st analgesia. Figure S8. Meta‐analysis of the rate of postoperative complications. Table S1. Detailed search strategy for each database. Table S2. List of excluded studies during the full‐text screening step. Table S3. Detailed information regarding the assessment tool and pain control protocol for each trial. [file OTO2-9-e70166-s001.docx]

**SUPPLEMENTAL TABLE AND FIGURE LEGENDS**

**Table S1.** Detailed search strategy for each database.

**Table S2.** List of excluded studies during the full-text screening step.

**Table S3.** Detailed information regarding the assessment tool and pain control protocol for each trial.

**Figure S1. (A)** Leave-one-out sensitivity analysis; **(B)** Subgroup analysis based on the use of adrenaline for the mean postoperative pain score at 1 hour.

**Figure S2. (A)** Leave-one-out sensitivity analysis; **(B)** Subgroup analysis based on the use of adrenaline for the mean postoperative pain score at 2 hours.

**Figure S3. (A)** Leave-one-out sensitivity analysis; **(B)** Subgroup analysis based on the use of adrenaline the mean postoperative pain score at 4 hours.

**Figure S4. (A)** Leave-one-out sensitivity analysis; **(B)** Subgroup analysis based on the use of adrenaline for the mean postoperative pain score at 6-8 hours.

**Figure S5. (A)** Leave-one-out sensitivity analysis; **(B)** Subgroup analysis based on the use of adrenaline for the mean postoperative pain score at 12 hours.

**Figure S6. (A)** Leave-one-out sensitivity analysis; **(B)** Subgroup analysis based on the use of adrenaline for the mean postoperative pain score at 24 hours.

**Figure S7. (A)** Leave-one-out sensitivity analysis; **(B)** Subgroup analysis based on the use of adrenaline for the mean score of time to 1^st^ analgesia.

**Figure S8.** Meta-analysis of the rate of postoperative complications.

| **Table S1.** Detailed search strategy for each database. | | | |
| --- | --- | --- | --- |
| **Database** | **Search Strategy** | **Filter** | **Results** |
| **PubMed** | (tonsillectom* OR adenotonsillectom* OR “tonsil surgery” OR “tonsil removal” OR “tonsillar surgery” OR “tonsillar removal”) AND ("Ropivacaine" OR “ropivacain*” OR “ropivacaine hydrochloride” OR "1-Propyl-2',6'-pipecoloxylidide" OR "1 Propyl 2',6' pipecoloxylidide" OR "Naropin" OR "Ropivacaine Monohydrochloride" OR "Ropivacaine Hydrochloride" OR "AL 381" OR "AL-381" OR "AL381" OR "Naropeine" OR "LEA 103" OR "LEA-103" OR "LEA103" OR "Ropivacaine Monohydrochloride, (S)-isomer" OR "1 propyl 2', 6' pipecoloxylidide" OR "hr 18034" OR "hr18034" OR "n (2, 6 dimethylphenyl) 1 propyl 2 piperidinecarboxamide" OR "n (2, 6 dimethylphenyl) 1 propylpipecolamide"[TW] OR "n (2, 6 dimethylphenyl) 1 propylpiperidine 2 carboxamide" OR "narop" OR "naropein" OR "naropin polyamp" OR "naropin sdv" OR "naropina" OR "noropine" OR "ropivacaina" OR "ropivacaine hydrochloride monohydrate" OR "tlc 590" OR "tlc590") AND ("Bupivacaine" OR "1-Butyl-N-(2,6-dimethylphenyl)-2-piperidinecarboxamide" OR "Marcain" OR "Bupivacain Janapharm" OR "Bupivacain-RPR" OR "Bupivacain RPR" OR "Bupivacaina Braun" OR "Carbostesin" OR "Bupivacaine Carbonate" OR "Bupivacaine Hydrochloride" OR "Bupivacaine Monohydrochloride, Monohydrate" OR "Buvacaina" OR "Dolanaest" OR "Sensorcaine" OR "Svedocain Sin Vasoconstr" OR "Marcaine" OR "Bupivacaine Anhydrous" OR "1 butyl 2', 6' pipecoloxylidide" OR "1 butyl n (2, 6 dimethylphenyl) 2 piperidinecarboxamide" OR "1 butyl n (2, 6 dimethylphenyl) piperidine 2 carboxamide" OR "anekain" OR "bicain" OR "bipuvacaine" OR "bucaine" OR "bupicaina" OR "bupinex" OR "bupirop" OR "bupirop simple sin preservantes" OR "bupisen" OR "bupivacain" OR "bupivacaine hydrochloride kit" OR "bupivacaine hydrochloride preservative free" OR "bupivacaine liposome" OR "bupivan" OR "bupivicaine" OR "bupizenge" OR "buvacainas" OR "buvasin" OR "chirocaina" OR "eladur" OR "exparel" OR "inibsa" OR "kamacaine" OR "lac 43" OR "lac43" OR "macaine" OR "marcaina" OR "marcaine hcl" OR "marcaine hydrochloride" OR "marcaine hydrochloride preservative free" OR "marcaine plain" OR "marcaine spinal" OR "optesia" OR "picain" OR "posidur" OR "posimir" OR "senpivac" OR "sensocaine" OR "sensorcaine-mpf" OR "sensoricaine" OR "sky 0302" OR "sky 0402" OR "sky0302" OR "sky0402" OR "xaracoll") | All Fields | N = 9 |
| **CENTRAL** | (tonsillectom* OR adenotonsillectom* OR “tonsil surgery” OR “tonsil removal” OR “tonsillar surgery” OR “tonsillar removal”) AND ("Ropivacaine" OR “ropivacain*” OR “ropivacaine hydrochloride” OR "1-Propyl-2',6'-pipecoloxylidide" OR "1 Propyl 2',6' pipecoloxylidide" OR "Naropin" OR "Ropivacaine Monohydrochloride" OR "Ropivacaine Hydrochloride" OR "AL 381" OR "AL-381" OR "AL381" OR "Naropeine" OR "LEA 103" OR "LEA-103" OR "LEA103" OR "Ropivacaine Monohydrochloride, (S)-isomer" OR "1 propyl 2', 6' pipecoloxylidide" OR "hr 18034" OR "hr18034" OR "n (2, 6 dimethylphenyl) 1 propyl 2 piperidinecarboxamide" OR "n (2, 6 dimethylphenyl) 1 propylpipecolamide"[TW] OR "n (2, 6 dimethylphenyl) 1 propylpiperidine 2 carboxamide" OR "narop" OR "naropein" OR "naropin polyamp" OR "naropin sdv" OR "naropina" OR "noropine" OR "ropivacaina" OR "ropivacaine hydrochloride monohydrate" OR "tlc 590" OR "tlc590") AND ("Bupivacaine" OR "1-Butyl-N-(2,6-dimethylphenyl)-2-piperidinecarboxamide" OR "Marcain" OR "Bupivacain Janapharm" OR "Bupivacain-RPR" OR "Bupivacain RPR" OR "Bupivacaina Braun" OR "Carbostesin" OR "Bupivacaine Carbonate" OR "Bupivacaine Hydrochloride" OR "Bupivacaine Monohydrochloride, Monohydrate" OR "Buvacaina" OR "Dolanaest" OR "Sensorcaine" OR "Svedocain Sin Vasoconstr" OR "Marcaine" OR "Bupivacaine Anhydrous" OR "1 butyl 2', 6' pipecoloxylidide" OR "1 butyl n (2, 6 dimethylphenyl) 2 piperidinecarboxamide" OR "1 butyl n (2, 6 dimethylphenyl) piperidine 2 carboxamide" OR "anekain" OR "bicain" OR "bipuvacaine" OR "bucaine" OR "bupicaina" OR "bupinex" OR "bupirop" OR "bupirop simple sin preservantes" OR "bupisen" OR "bupivacain" OR "bupivacaine hydrochloride kit" OR "bupivacaine hydrochloride preservative free" OR "bupivacaine liposome" OR "bupivan" OR "bupivicaine" OR "bupizenge" OR "buvacainas" OR "buvasin" OR "chirocaina" OR "eladur" OR "exparel" OR "inibsa" OR "kamacaine" OR "lac 43" OR "lac43" OR "macaine" OR "marcaina" OR "marcaine hcl" OR "marcaine hydrochloride" OR "marcaine hydrochloride preservative free" OR "marcaine plain" OR "marcaine spinal" OR "optesia" OR "picain" OR "posidur" OR "posimir" OR "senpivac" OR "sensocaine" OR "sensorcaine-mpf" OR "sensoricaine" OR "sky 0302" OR "sky 0402" OR "sky0302" OR "sky0402" OR "xaracoll") | All text | N = 19 |
| **Web of Science** | (tonsillectom* OR adenotonsillectom* OR "tonsil surgery" OR "tonsil removal" OR "tonsillar surgery" OR "tonsillar removal") AND (ropivacain* OR "ropivacaine hydrochloride" OR "ropivacaine monohydrochloride" OR naropeine OR naropin OR "LEA 103" OR "LEA-103" OR "AL 381" OR "1 Propyl 2',6' pipecoloxylidide" OR "(S)-Ropivacaine" OR "84057-95-4" OR "rocaine" OR "local anaesthesia" OR "local anesthesia" OR "local analgesia" OR "local anesthetic" OR "local anaesthetic") AND ("Bupivacaine" OR "1-Butyl-N-(2,6-dimethylphenyl)-2-piperidinecarboxamide" OR "Marcain" OR "Bupivacain Janapharm" OR "Bupivacain-RPR" OR "Bupivacain RPR" OR "Bupivacaina Braun" OR "Carbostesin" OR "Bupivacaine Carbonate" OR "Bupivacaine Hydrochloride" OR "Bupivacaine Monohydrochloride, Monohydrate" OR "Buvacaina" OR "Dolanaest" OR "Sensorcaine" OR "Svedocain Sin Vasoconstr" OR "Marcaine" OR "Bupivacaine Anhydrous”) | All Fields | N = 115 |
| **Scopus** | (tonsillectom* OR adenotonsillectom* OR "tonsil surgery" OR "tonsil removal" OR "tonsillar surgery" OR "tonsillar removal") AND (ropivacain* OR "ropivacaine hydrochloride" OR "ropivacaine monohydrochloride" OR naropeine OR naropin OR "LEA 103" OR "LEA-103" OR "AL 381" OR "1 Propyl 2',6' pipecoloxylidide" OR "(S)-Ropivacaine" OR "84057-95-4" OR "rocaine" OR "local anaesthesia" OR "local anesthesia" OR "local analgesia" OR "local anesthetic" OR "local anaesthetic") AND ("Bupivacaine" OR "1-Butyl-N-(2,6-dimethylphenyl)-2-piperidinecarboxamide" OR "Marcain" OR "Bupivacain Janapharm" OR "Bupivacain-RPR" OR "Bupivacain RPR" OR "Bupivacaina Braun" OR "Carbostesin" OR "Bupivacaine Carbonate" OR "Bupivacaine Hydrochloride" OR "Bupivacaine Monohydrochloride, Monohydrate" OR "Buvacaina" OR "Dolanaest" OR "Sensorcaine" OR "Svedocain Sin Vasoconstr" OR "Marcaine" OR "Bupivacaine Anhydrous”) | Article title, Abstract, Keywords | N = 118 |
| **Google Scholar** | (tonsillectomy AND ropivacaine AND bupivacaine) | All Fields | N = 100 (first 5 pages) |

| **Table S2.** List of excluded studies during the full-text screening step. | | |
| --- | --- | --- |
| **Study ID** | **Title** | **Reason of exclusion** |
| **Deng et al. 2024** | Comparative Effectiveness of Analgesia for Early Pain Management After Pediatric Tonsillectomy: A Systematic Review and Network Meta-Analysis | Review Article |
| **Kim et al. 2023** | The efficacy and safety of peri-tonsillar administrated agents on pain treatment after pediatric tonsillectomy: A network meta-analysis | Review Article |
| **Arikan et al. 2008** | High-dose ropivacaine versus bupivacaine for posttonsillectomy pain relief in adults | Adults’ population |
| **Park et al. 2007** | The effects of glossopharyngeal nerve block on postoperative pain relief after tonsillectomy: the importance of the extent of obtunded gag reflex as a clinical indicator | Glossopharyngeal nerve block (GNB) |
| **Hollis et al. 1999** | Perioperative local anaesthesia for reducing pain following tonsillectomy | Review Article |
| **Aydin et al. 2007** | Effect of perioperative administration of ropivacaine or bupivacaine on postoperative pediatric adenotonsillectomy pain and diet | Conference abstract |
| **Kurtipek et al. 2004** | Comparison of peritonsiller infiltration with bupivacaine and ropivacaine on postoperative pain after tonsillectomy in paediatric patients A-617 | Conference abstract |
| **Stelter et al. 2010** | Comparison of two different local anaesthetic infiltrations for postoperative pain relief in tonsillectomy: a prospective, randomised, double blind, clinical trial | Not compared with ropivacaine |
| **Sadanandan et al. 2025** | Efficacy of Levobupivacaine versus Ropivacaine for Tonsillar Pillar Block in Patients Undergoing Tonsillectomy: A Randomised Clinical Trial | Not compared with bupivacaine |
| **Junaid et al. 2020** | Intraoperative use of analgesics in tonsillar fossa and postoperative evaluation with visual analogue scale scores—A prospective, randomized, placebo-controlled, double-blind clinical trial | Not compared with ropivacaine |
| **Nitin et al. 2023** | To Compare the Effects of Post‑tonsillectomy Intra‑operative Infiltration of Ropivacaine Versus Bupivacaine in Tonsillar Fossa | Adults’ population |
| **Nasser et al. 2023** | Ropivacaine 0.25% versus bupivacaine 0.25% in immediate post tonsillectomy pain management: a randomised control trial | Adults’ population |
| **Hydri et la. 2024** | Ropivacaine versus Bupivacaine post-tonsillectomy pain relief perspective | Adults’ population |

| **Table S3.** Detailed information regarding the pain assessment tool and pain control protocol for each trial. | | | |
| --- | --- | --- | --- |
| **Study ID** | **Pain assessment tool** | **Number of points** | **Pain control protocol** |
|  |  |  |  |
| **Akoglu 2006** | Modified Children’s Hospital of Eastern Ontario Pain Scale (mCHEOPS) | 10-point | If the pain score was greater than 5, a rescue medication including fentanyl citrate 0.5 mg/kg, or oral acetaminophen 10 mg/kg was given. |
| **Gudi 2014** | Visual Analog Scale (VAS) | 10-point | For post operative pain Inj. Diclofenac Sodium 1mg/kg iv was given to patients who complained of severe pain and syrup combiflam thrice daily were given to patients for 5 days in the post operative period. |
| **Kumar 2021** | Modified Children’s Hospital of Eastern Ontario Pain Scale (mCHEOPS) | 10-point | If the pain score is greater than 5, a rescue medication including fentanyl citrate 1 mcg/kg will be administered, and when patients tolerated fluids, oral acetaminophen 10 mg/kg will be given. |
| **Mehta 2019** | Wong Baker Face Scale (WBFS) | 10-point | Rescue analgesics included acetaminophen in dose of 10 mg/kg per dose, subject to a maximum of 4 gm. |
| **Özkıriş 2012** | Visual analog pain scale (VAS) | 10-point | All patients were administered post-operatively oral paracetamol pediatric suspension (40 mg/kg per day). |
| **Rao 2022** | Children's Hospital of Eastern Ontario Pain Scale (CHEOPS) | 13-point | Rescue dose of fentanyl was given if pain score was above 5. |
| **Unal 2007** | Visual analog pain scale (VAS) | 10-point | A 5 mg kg1 acetaminophen (oral route) given at VAS was higher than 4 |

**Figure S1. (A)** Leave-one-out sensitivity analysis; **(B)** Subgroup analysis based on the use of adrenaline for the mean postoperative pain score at 1 hour.

**(A)**

**
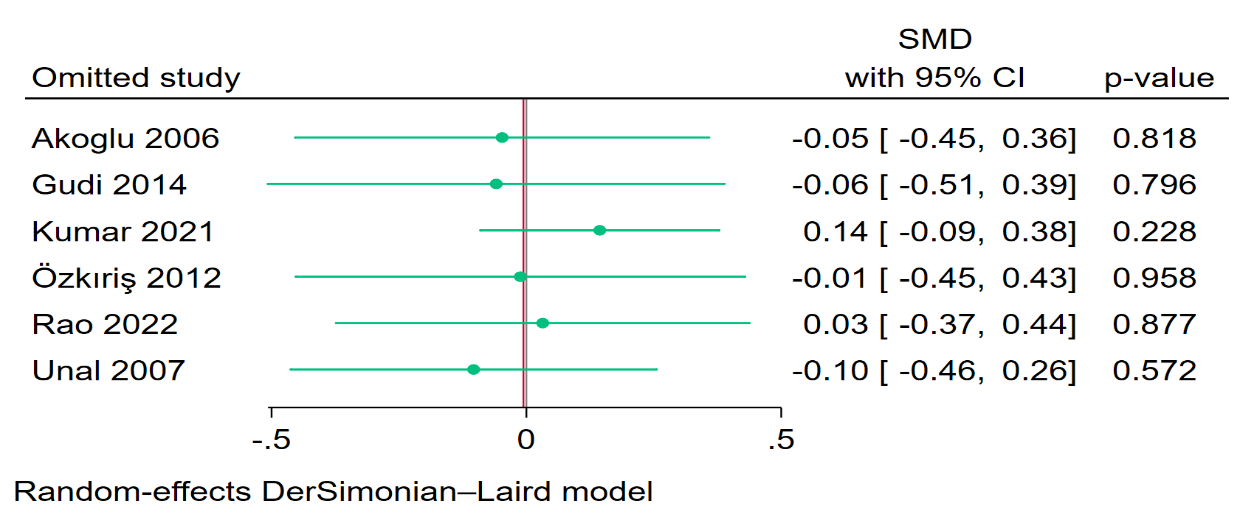
**

**(B)**

**
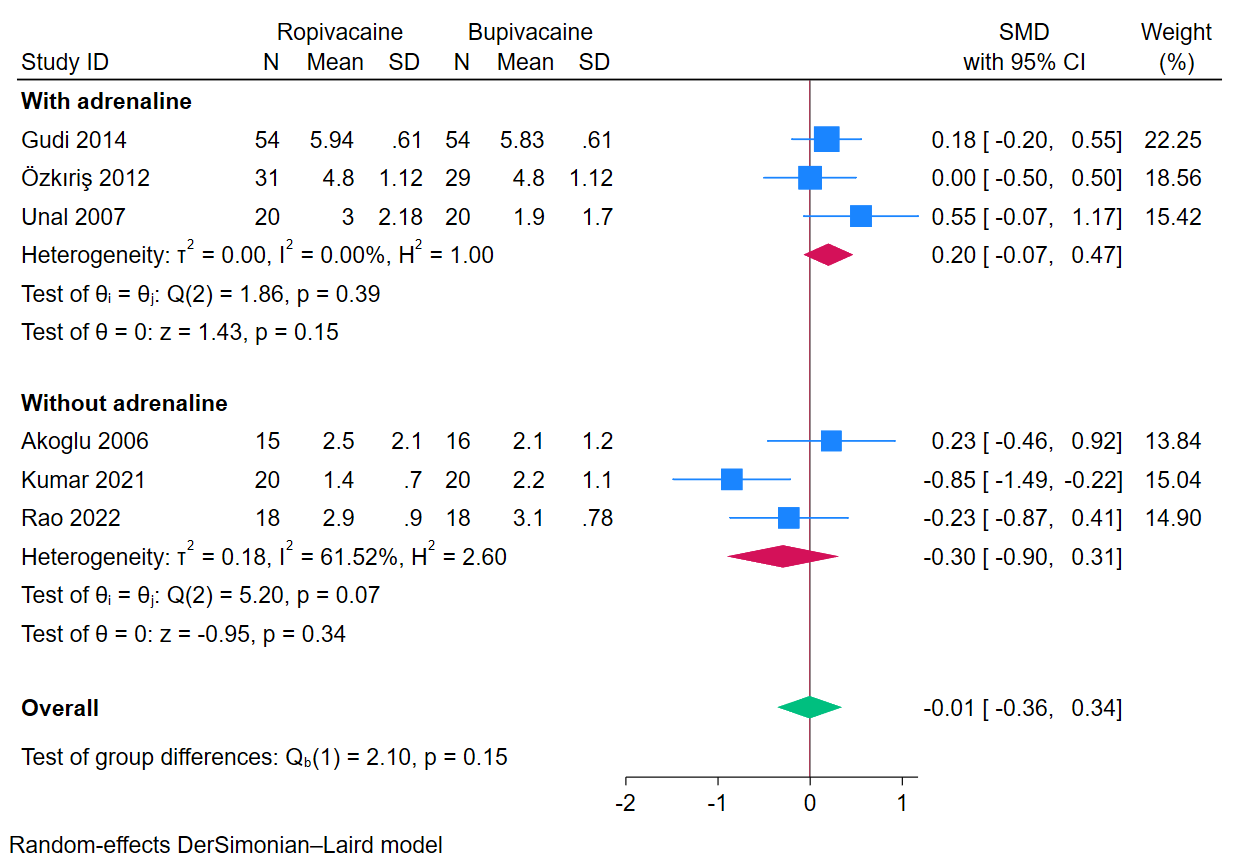
**

**Figure S2. (A)** Leave-one-out sensitivity analysis; **(B)** Subgroup analysis based on the use of adrenaline for the mean postoperative pain score at 2 hours.

**(A)**

**
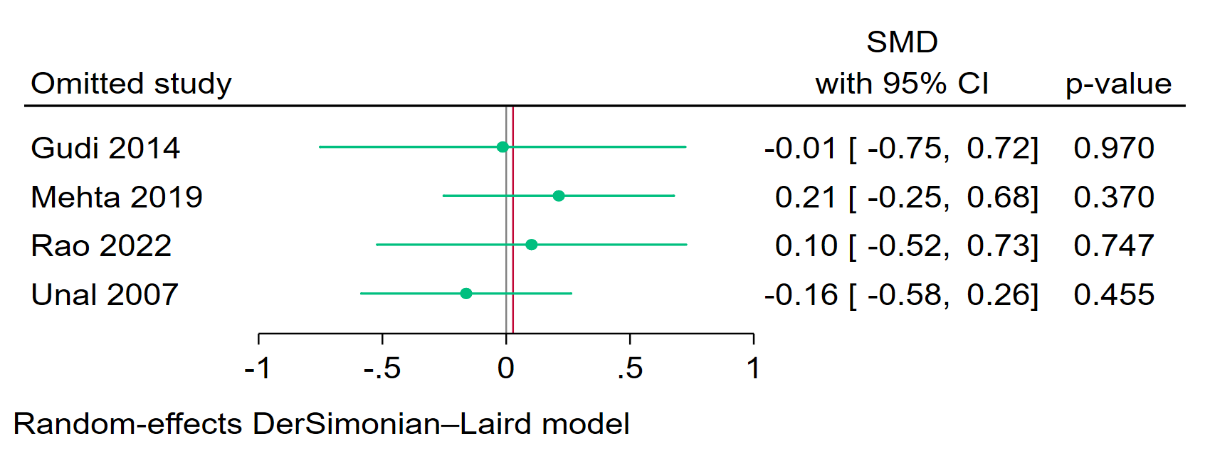
**

**(B)**

**
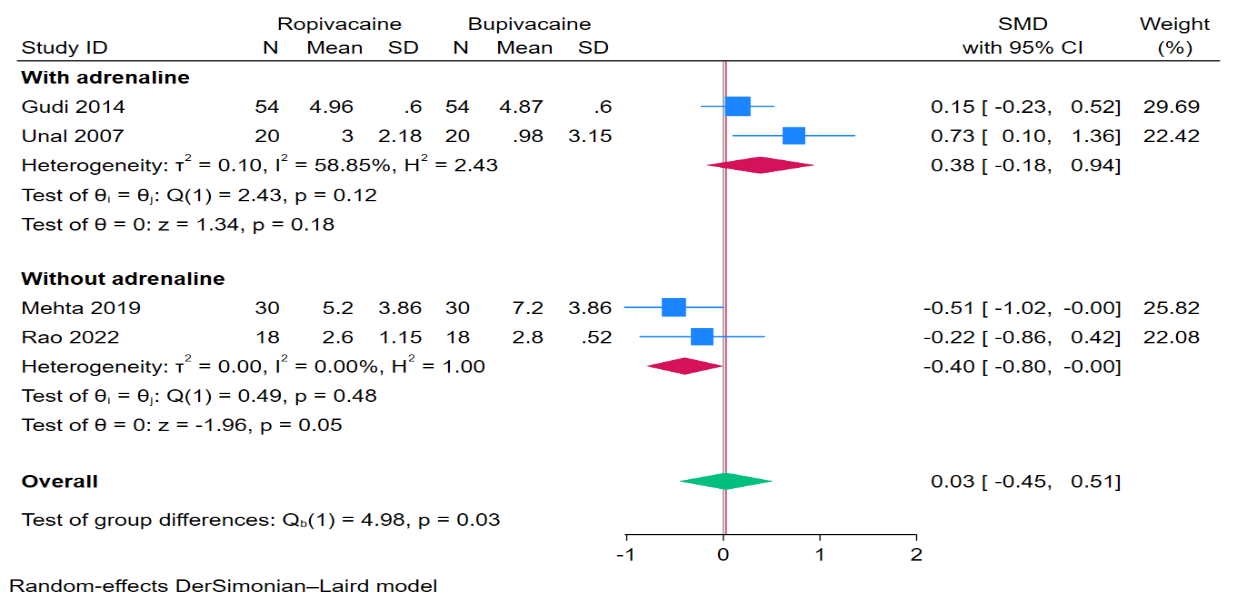
**

**Figure S3. (A)** Leave-one-out sensitivity analysis; **(B)** Subgroup analysis based on the use of adrenaline for the mean postoperative pain score at 4 hours.

**(A)**

**
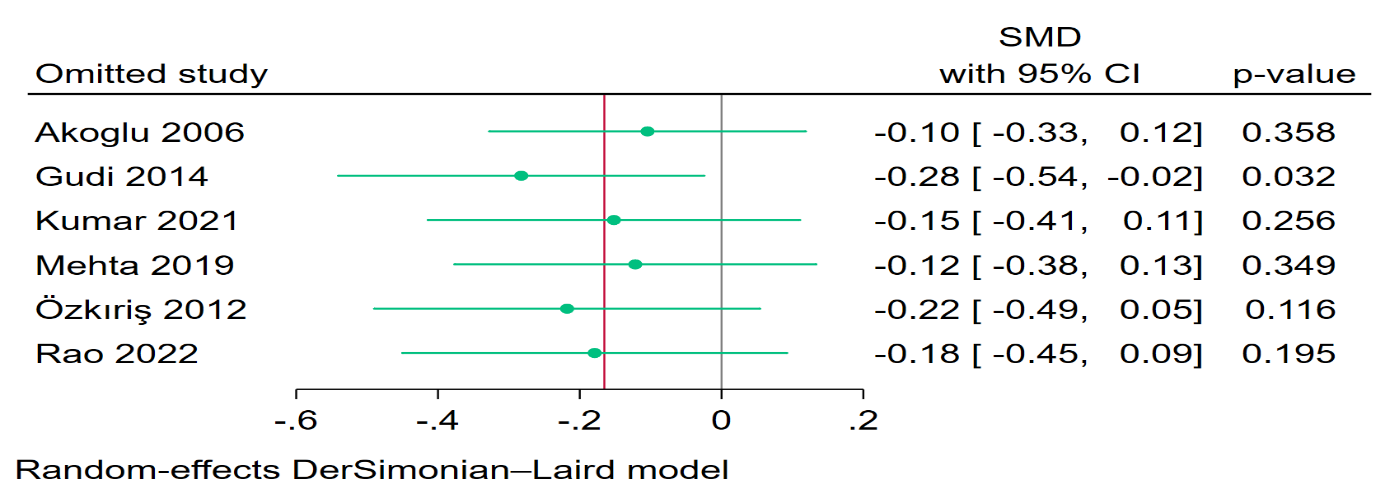
**

**(B)**

**
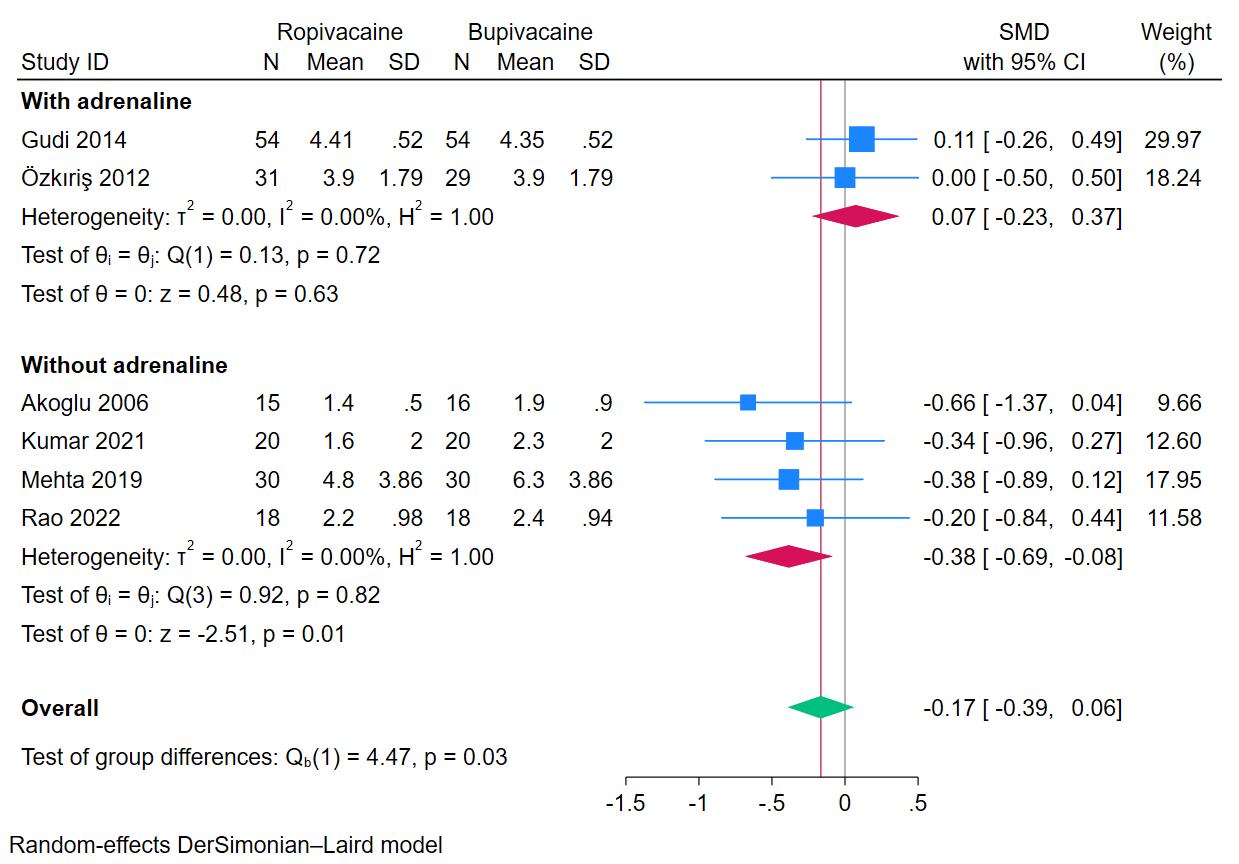
**

**Figure S4. (A)** Leave-one-out sensitivity analysis; **(B)** Subgroup analysis based on the use of adrenaline for the mean postoperative pain score at 6-8 hours.

**(A)**

**
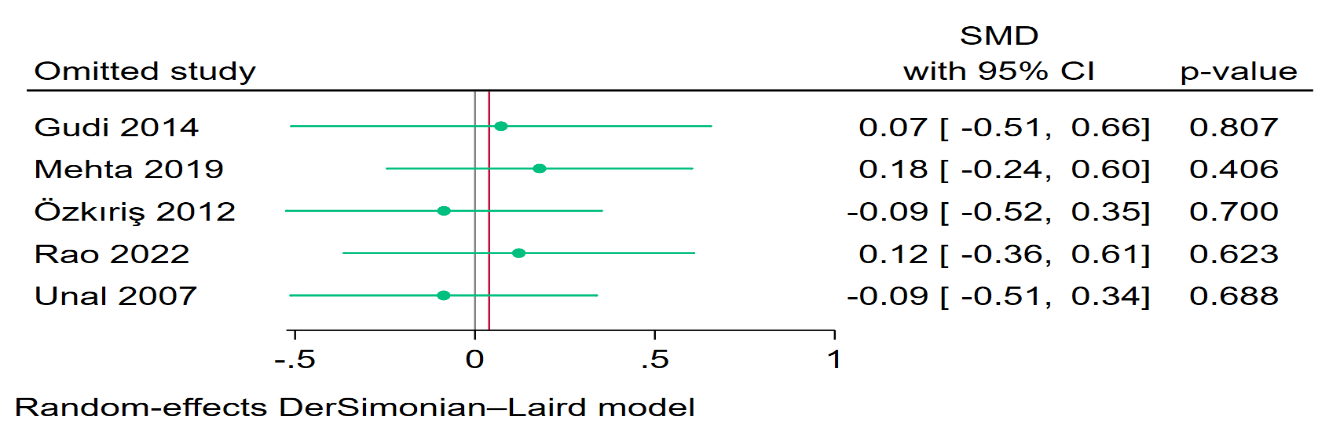
**

**(B)**

**
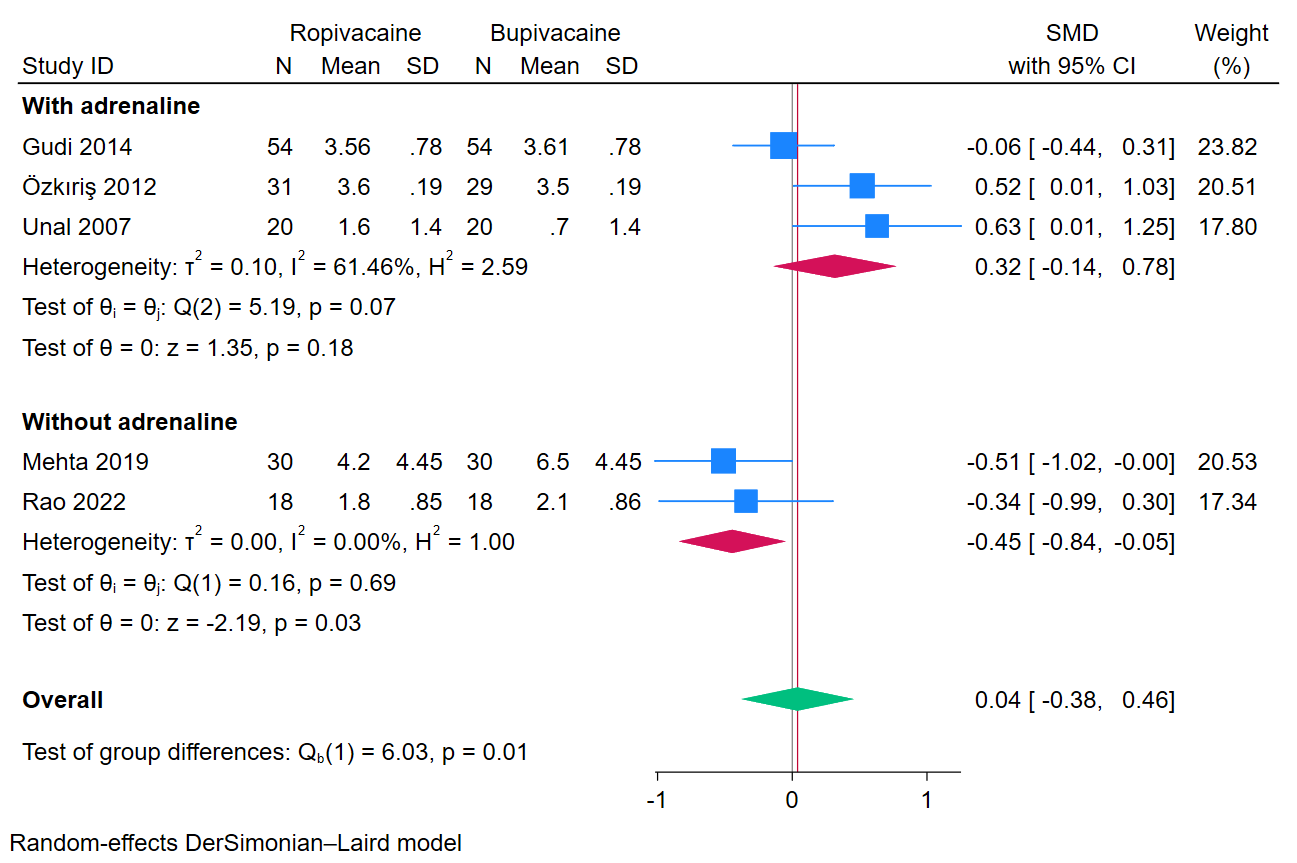
**

**Figure S5. (A)** Leave-one-out sensitivity analysis; **(B)** Subgroup analysis based on the use of adrenaline for the mean postoperative pain score at 12 hours.

**(A)**

**
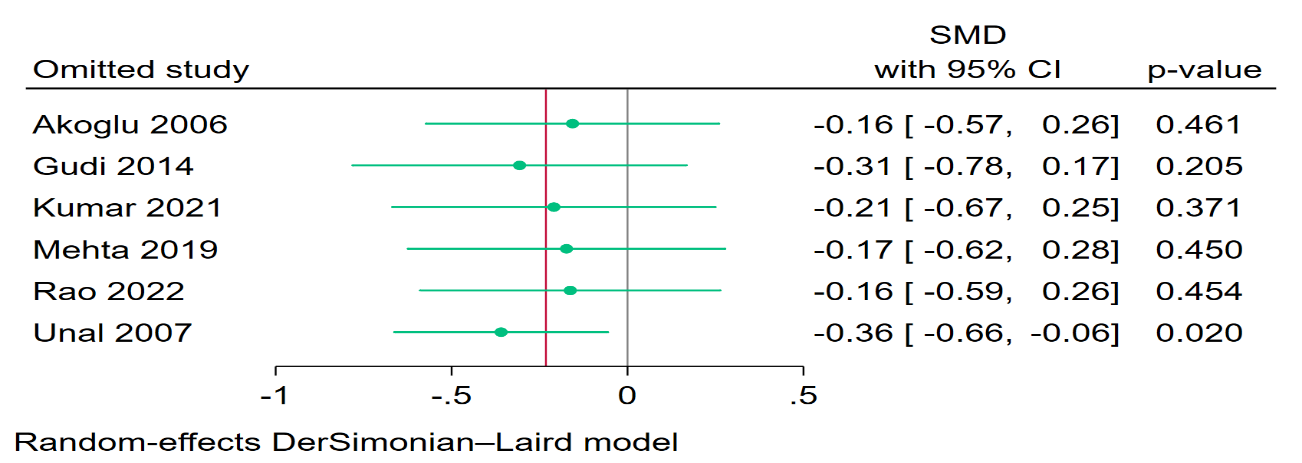
**

**(B)**

**
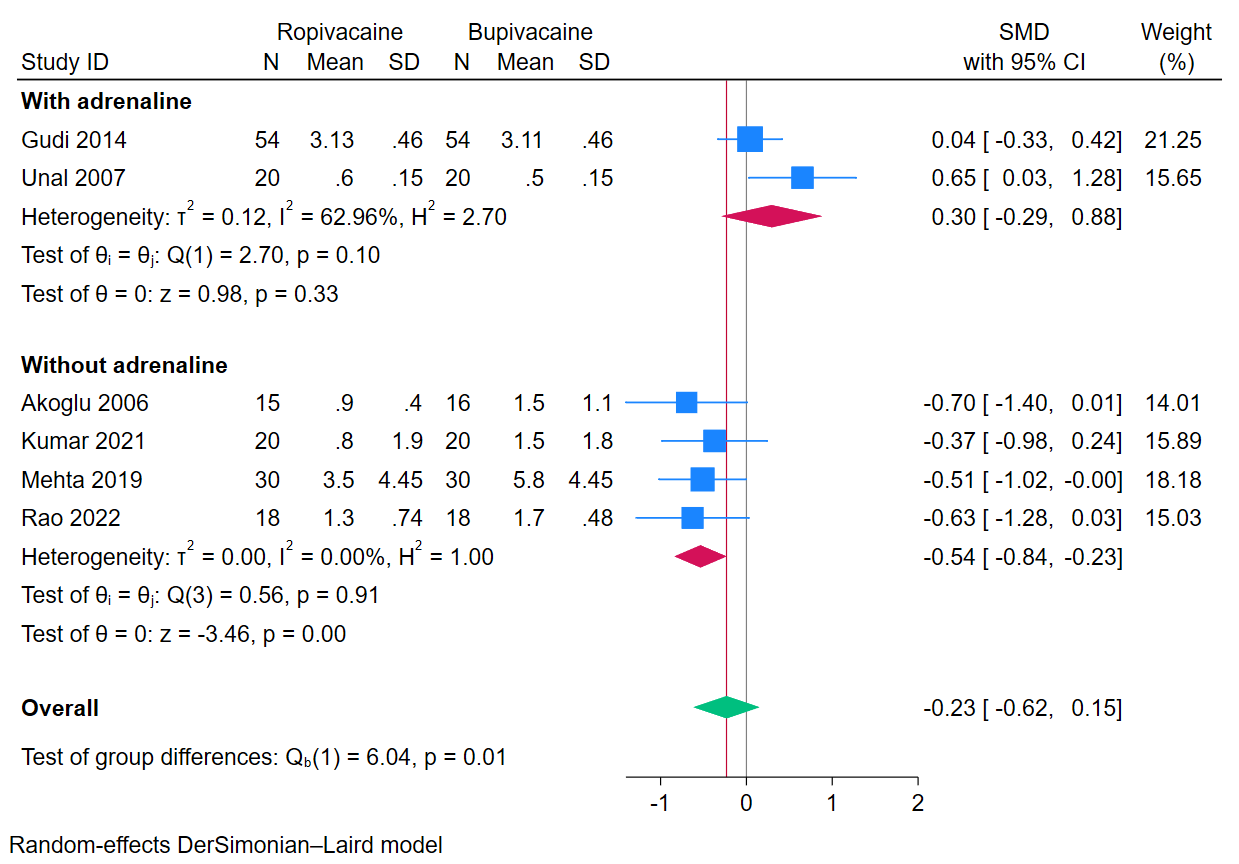
**

**Figure S6. (A)** Leave-one-out sensitivity analysis; **(B)** Subgroup analysis based on the use of adrenaline for the mean postoperative pain score at 24 hours.

**(A)**

**
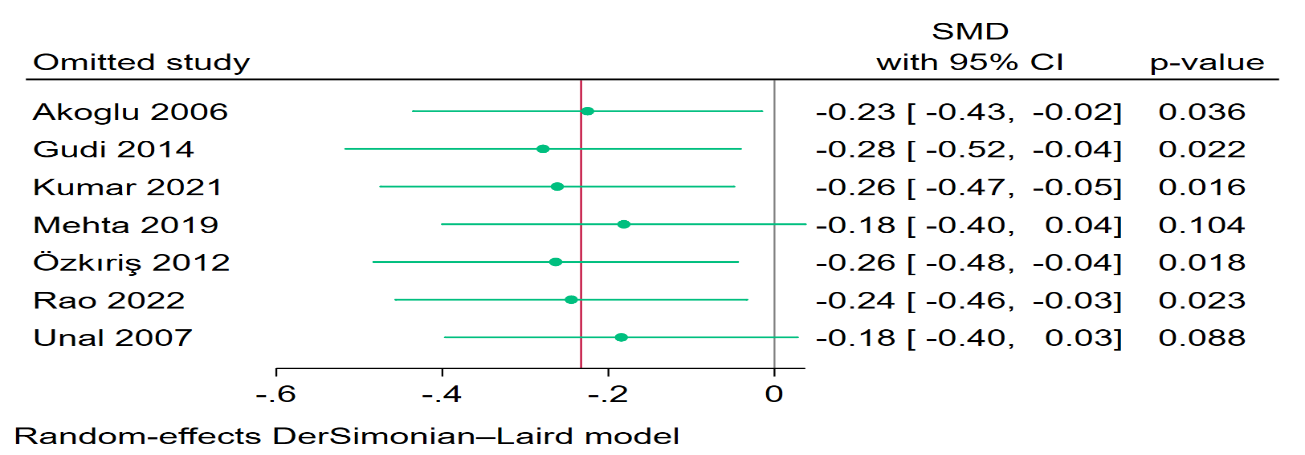
**

**(B)**

**
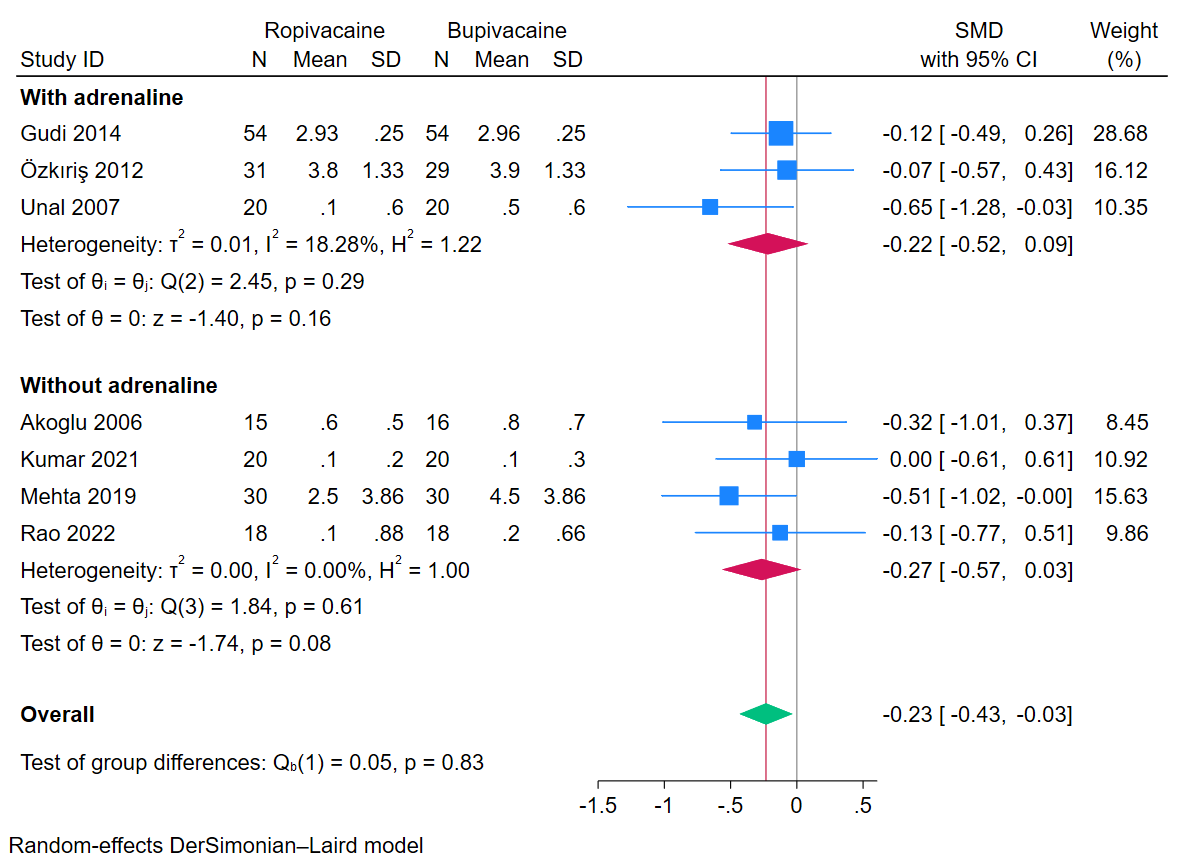
**

**Figure S7. (A)** Leave-one-out sensitivity analysis; **(B)** Subgroup analysis based on the use of adrenaline for the mean score of time to 1^st^ analgesia.

**(A)**

**
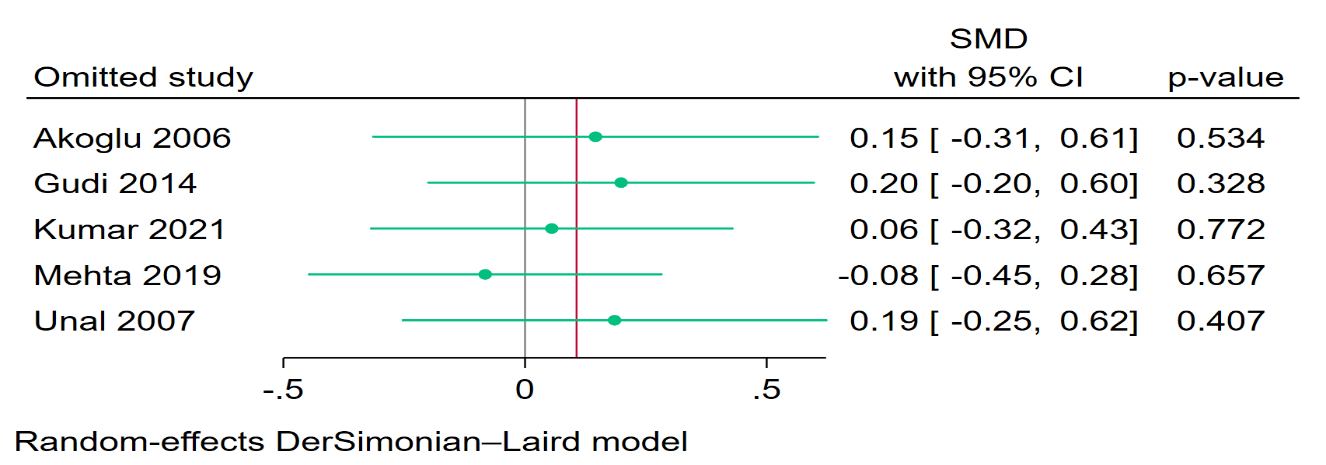
**

**(B)**

**
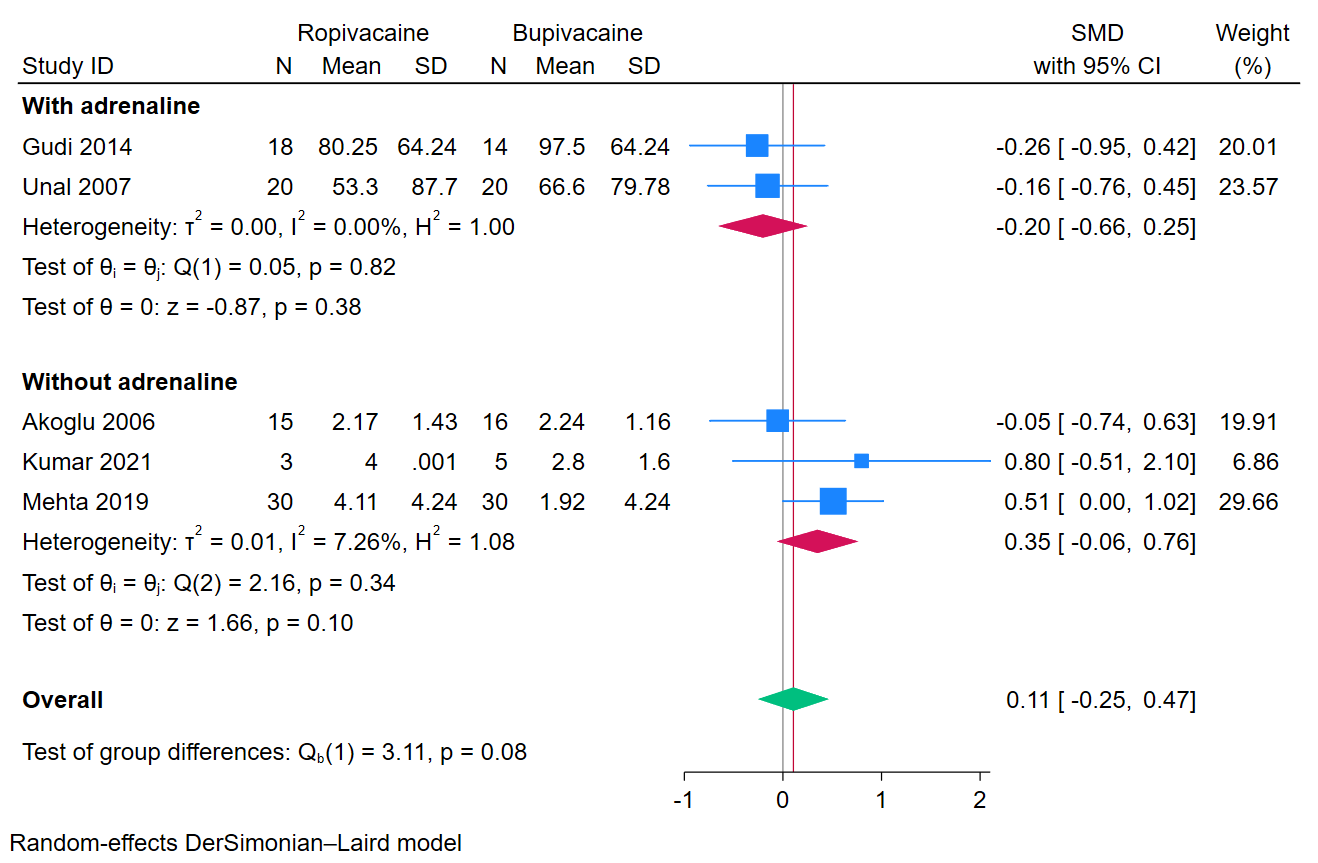
**

**Figure S8.** Meta-analysis of the rate of postoperative complications.

**
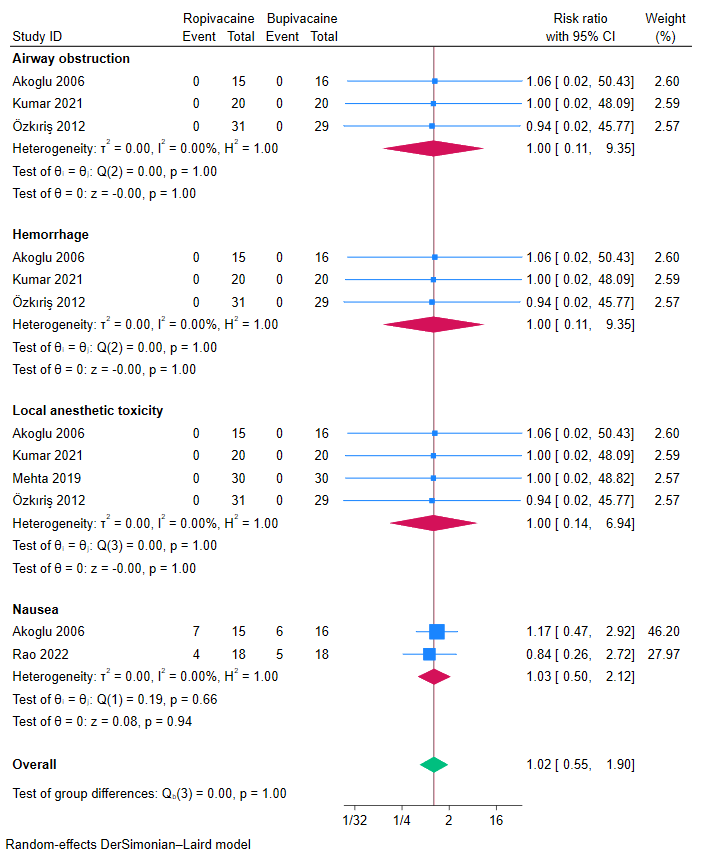
**
